# End-to-End Backbone Cyclization Enhances Passive Permeability of bRo5 Oligomeric Depsipeptides with Nonlinear Size Dependence

**DOI:** 10.1021/acsmedchemlett.5c00037

**Published:** 2025-03-20

**Authors:** Madelaine P. Thorpe, Corey R. Hopkins, Jeffrey N. Johnston

**Affiliations:** † Department of Chemistry and Vanderbilt Institute of Chemical Biology, 5718 Vanderbilt University, Nashville, Tennessee 37235, United States; ‡ Department of Pharmaceutical Sciences, College of Pharmacy, 12284 University of Nebraska Medical Center, Omaha, Nebraska 68198, United States

**Keywords:** permeability, macrocyclization, cyclic depsipeptide, bRo5 peptide, peptide bioavailability

## Abstract

A majority of drugs are small molecules that satisfy Lipinski’s Rule-of-Five (Ro5), but efforts to target topologically complex biomolecular interactions have reignited interest in nonconforming molecular therapeutics, dubbed “beyond Ro5 (bRo5)”. Broadly useful design principles for bRo5 molecules are few in number, although several studies have highlighted the benefit to bioavailability and proteolytic stability that can result from the introduction of a constraining ring into conformationally mobile peptides. Here we show that a linear oligomeric depsipeptide (OD) template can be leveraged to link size to permeability, while the corresponding cyclic oligomeric depsipeptide (COD) series is used to determine the impact of cyclization as an added conformational constraint. Unexpectedly, certain macrocycle sizes confer a greater benefit to permeability than others.

## Graphical abstract

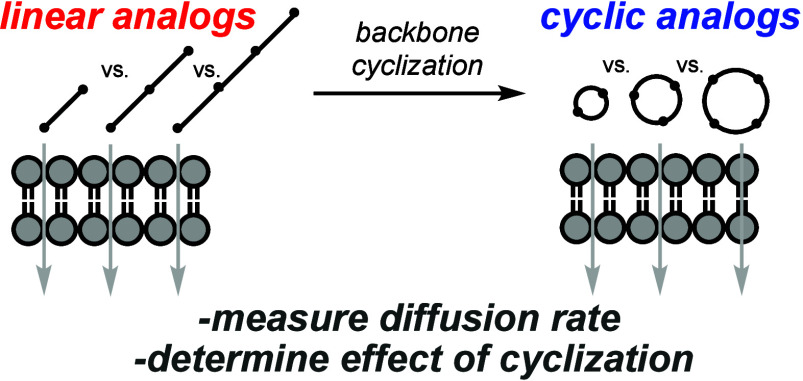

There are up to 650,000 undruggable protein–protein interactions in the human interactome, ranging widely in complexity and size. Extracellular or cell surface targets may prove to be “druggable” with larger molecules such as antibodies or biologics.^1,2^ Use of relatively large complex molecules can address target engagement, but the design of membrane-permeable variants to engage intracellular targets is a widely accepted challenge. As nontraditional “beyond Rule of 5” (bRo5) molecules, peptides and their analogs have emerged as tools capable of selectively engaging complex targets when small molecules with conventional drug-like features have failed.^3^ Design principles that endow bRo5 molecules with traditional drug properties have developed slowly, while the popularity of cyclic peptidic compounds as promising leads in drug discovery has increased. There are currently 80 peptides that are marketed drugs.^3^ Peptide macrocyclization^4–7^ has emerged as the most common tactic to enhance pharmacological properties within this group, replicating a drug development tactic^8^ and feature of many naturally occurring bioactive peptides.^9^

Within a broader program to develop antiarrhythmic agents,^10,11^ we speculated that the cyclic feature of the hit compound *ent*-verticilide might be critical to its apparent cell permeability. This suspicion was further fueled by the discovery of a second inhibitor, *ent*-verticilide B1, a cyclic oligomer of *ent*-verticilide (**1**).^12^ Many reports have examined structural modifications of peptides to increase permeability: side chain modifications,^13^ amide *N*-methylation,^14,15^ conversion of amide to thioamide,^16^ and residue–residue side-chain cyclization.^17^ The cyclization strategy to limit conformational mobility includes several tactics. Cyclization using the residues of two peptide side chains is the most common one that typically preserves the backbone hydrogen bond donors (HBDs). Ring closure can be achieved through various types of connectivity beyond side chain-to-side chain, including end-to-end (also known as backbone cyclization), head-to-side chain, and side chain-to-tail, commonly through an amide bond coupling reaction.^6,18^ Numerous methods to introduce rings into peptides have been used, typically leveraging existing functionality.^19^

To the best of our knowledge, no study has reported a systematic and rigorous analysis of the effect of size on passive diffusion while minimally affecting other structural and functional determinants of permeability.^20^ Lead optimization campaigns that introduce a ring system to restrict conformation are often binary experiments that compare a host of properties that include passive diffusion. Each case study is limited to a narrow molecular weight range, and side chains are not uniformly held constant. Therefore, we sought to design a more comprehensive series to probe the difference in passive diffusion of linear-to-cyclic (depsi)­peptides. *ent*-Verticilide and a complete set of 22 analogs between two series allowed precise investigation of the acyclic to cyclic transition to link molecular size (series 1) and backbone composition (series 2) to permeability (Figure 1), while holding constant the contribution of alkyl (pentyl and methyl) side chains. Quantifying changes in this manner can inform peptide SAR campaigns and lead to compound optimization.

**Figure 1:**
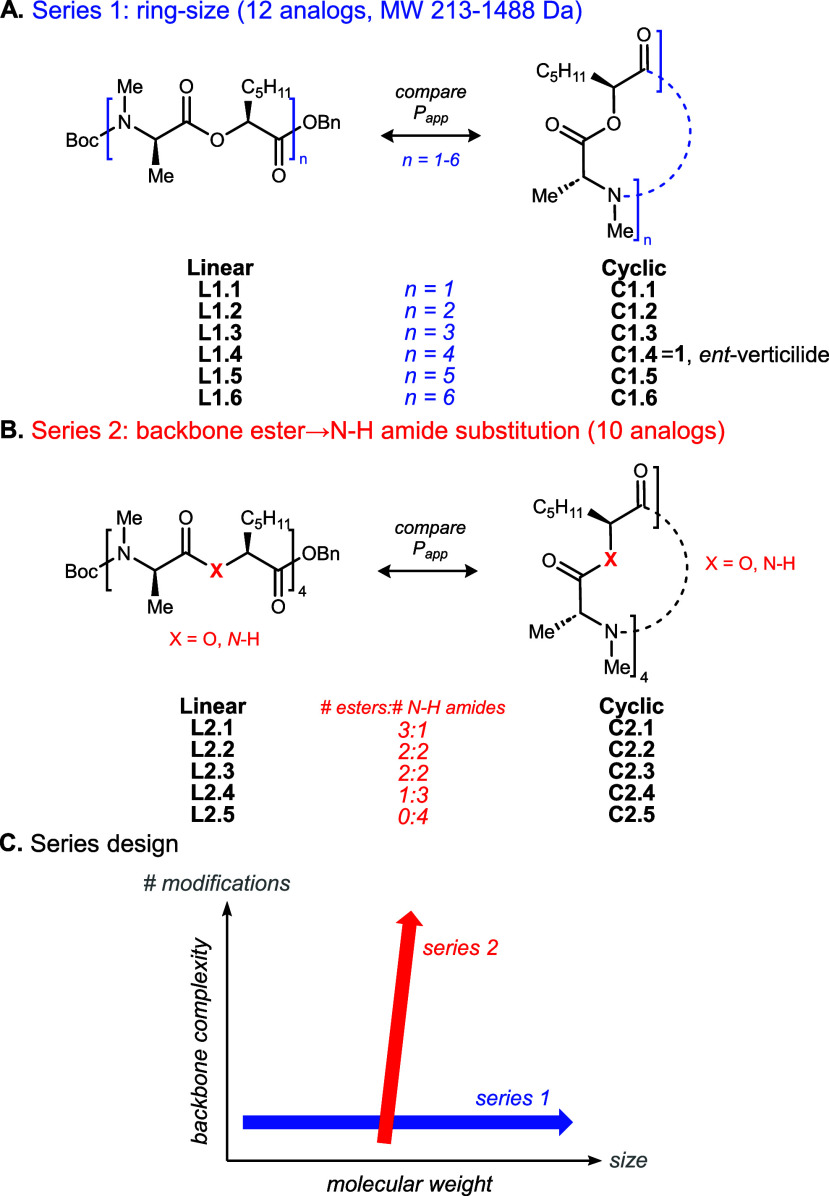
Determination of passive permeability for linear versus end-to-end forms of (depsi)­peptides: overview of series development to deconvolute size (series 1) and functionality (series 2) contributions.

Prior work established the permeability of **1** (=**C1.4**) by PAMPA to be similar to that of cyclosporin A.^15,21^ The oligomeric nature of **1** allows a single structural modification to be repeated systematically throughout the molecule and the opportunity to question how size contributes. The effect of size (backbone length) and end-to-end backbone cyclization on passive diffusion were probed first. For ease of reference, each analog is labeled as linear (L) or cyclic (C) alongside its compound number. Beginning with the smallest repeating unit (didepsipeptide, 6), chain lengths of 12, 18, 24, 30, and 36 were prepared, **(** Figure 1A, L1.1–1.6 and C1.1–1.6). Protection of each linear depsipeptide end with *N*-Boc and *O*-benzyl was held constant, while the ratio of side chains to backbone functionality was also maintained. For short oligomer lengths, the molecular weight change (ΔMW ∼ 208) is more pronounced between the protected linear and cyclic forms (i.e., L1.1 vs C1.1), while the difference at higher lengths is less (e.g., L1.5 vs C1.5). These analogs span a wide range of molecular weight (231–1488 Da), providing the opportunity to determine whether the *N*- and *C*- protecting groups drive permeability behavior. Permeability for series 1 compounds was measured by the PAMPA experiment (Table 1).^15^

**Table 1:**
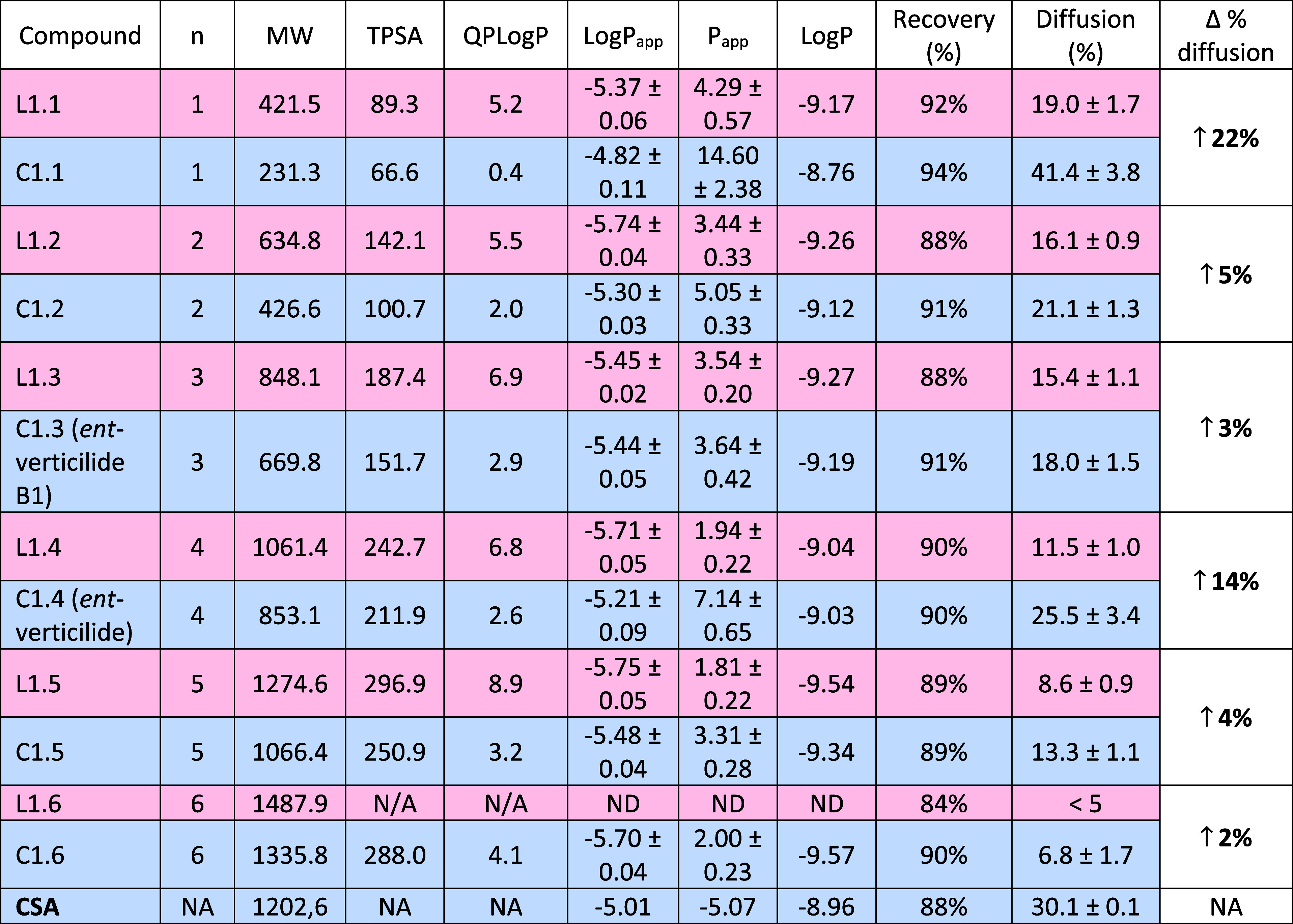
Calculated and Experimental Data for Series 1, Correlating Size and Permeability for Linear (L1.*x*) and Cyclic (C1.*x*) Depsipeptides^a^

a Entries are shaded with red = linear and blue = macrocyclic. Definitions: *n* = number of repeating monomer units, MW = molecular weight (g/mol), TPSA = topological polar surface area (Å), QPLogP = calculated octanol/water logP using Schrödinger QikProp, LogP_app_ and *P*_app_ = experimental diffusion coefficient determined from a ratio-based method given as mean ± standard deviation (×10^–6^ cm/s), LogP = determined from standard curve to measure [D] and [A], % diff (diffusion) = [acceptor]/[acceptor + donor], and recovery = (acceptor + donor)/total analyte, increase in % diffusion = difference between percent diffusion of macrocycle vs linear counterpart, ND = below limit of detection, N/A = molecular weight is too high for prediction using Schrödinger QikProp.

These results are displayed as a bar graph in Figure 2 with permeability (*P*_app_) plotted against depsipeptide backbone length. Focusing on the trend for linear oligomers, a clear decrease in permeability is observed as chain length increases, ending with no detectable permeability in the case of L1.6 (<5% diffusion). Oligomers L1.1, L1.2, and L1.3 exhibited similar levels of permeability, and L1.4 exhibited a significant decrease. End-to-end backbone cyclization enhanced permeability significantly at all oligomer lengths. A tremendous increase was observed for C1.1 and C1.4, while C1.2, C1.3, C1.5, and C1.6 exhibited smaller but significant increases relative to their linear counterparts. It is worth noting that the passive diffusion of L1.6 was below the limit of detection of 5%, while that of C1.6 was measured at 7% diffusion. In all cases, the percent recovery of analyte was >84%, with most averaging 90%.

**Figure 2:**
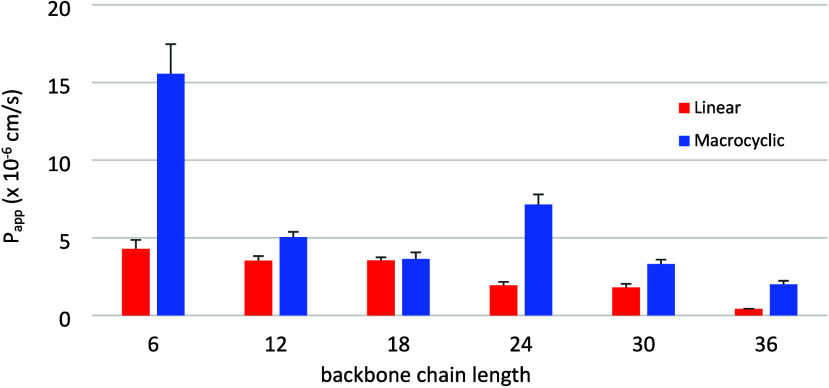
Effect of macrocyclization on passive membrane permeability. Linear analogs are in red, and macrocyclic analogs are in blue. Values shown are the mean ± standard deviation. Definitions: *P*_app_ = rate of diffusion across artificial membrane (cm/s), analog size = number of atoms in the backbone, backbone chain length: 6 = 1.1, 12 = 1.2, 18 = 1.3, 24 = 1.4, 30 = 1.5, 36 = 1.6.

Series 2 analogs differed minimally by molecular weight within each linear and cyclic subseries. Ring size and side chain lipophilicity were also unchanged, but the backbone polarity was modified by substituting esters for more polar N–H amides to complement the existing four *N*-Me amides (Figure 3). This systematic substitution of esters for N–H amides introduces hydrogen bond donors (HBDs). Changing the composition of the backbone in this manner increases polarity and was found to increase permeability in some cases by comparison to *ent*-verticilide.^15^ A key question was whether end-to-end ring formation would similarly confer increased permeability relative to chain forms, analogous to series 1. The analogs are organized by increasing substitution of oxygen for N–H amide (2.1–2.5). Analogs 2.2 and 2.3 are isomeric, differentiated by whether the N–H amides are adjacent (2.2) or alternating (2.3).^15^ It is worth noting that as a consequence of the synthesis, compound L2.4 was isolated as the free amine. Five linear analogs were prepared, each with sequential ester bond substitutions. Each of these linear precursors was then deprotected and cyclized to provide the macrocyclic forms.

**Figure 3:**
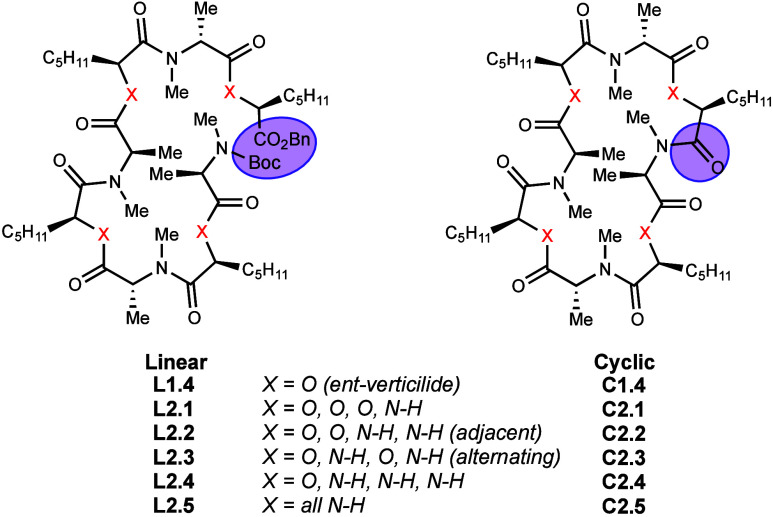
Structures of series 2 linear (**L*y***) and cyclic (**C*y***) analogs (X = O, N–H).

These results are displayed as a bar graph in Figure 4 with permeability (*P*_app_) plotted against series 2.1–2.5 analogs in sequence, with linear and cyclic forms listed side-by-side. As shown in Figure 4 changes in passive permeability were observed for all linear compounds, generally decreasing permeability with increasing N–H amide content, as expected. Compared to the parent compound with four esters, however, analogue 2.1 exhibited increased permeability despite the increase in polarity and hydrogen bonding capability associated with the exchange of one ester for an N–H amide. A decrease of permeability with increasing N–H amide content is also observed for the cyclic forms of each analog. The decreasing permeability is most significant between single- and double-exchange analogs (Figure 4, 2.1 vs 2.2, 2.3). The macrocyclic depsipeptides within this series show heightened permeability compared to their linear counterparts in all cases, much like series 1 trends. The difference was most pronounced in analogs 2.1 and 2.2. Significant increases were observed with 2.3, 2.4, and 2.5 (Table 2).

**Table 2:**
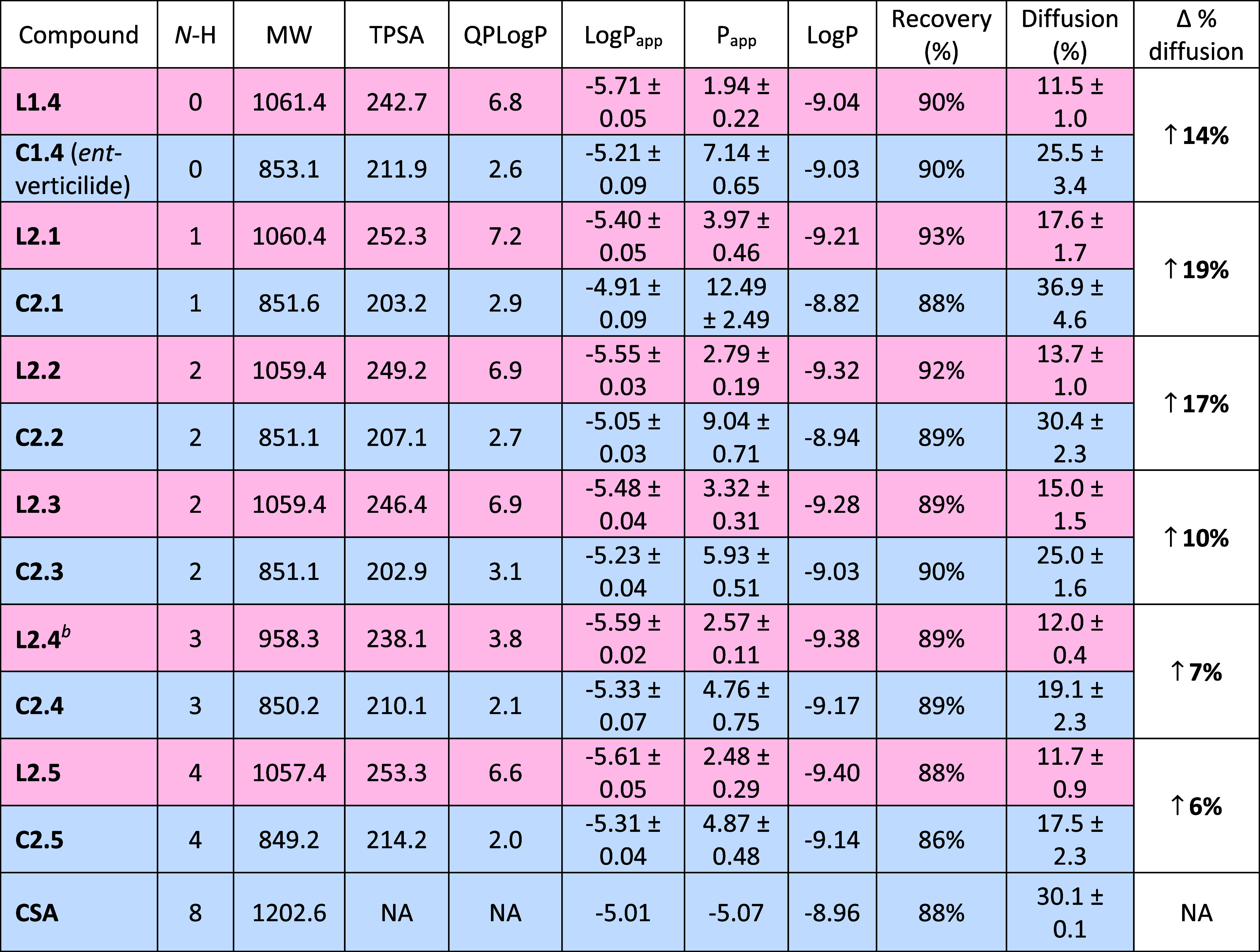
Calculated and Experimental Data for Series 2 Backbone-Modified Analogs^a^

a Entries are shaded with red = linear and blue = macrocyclic. Definitions: N–H = number of N–H amides in the backbone, MW = molecular weight (g/mol), TPSA = topological polar surface area (Å), QPLogP = calculated octanol/water logP using Schrödinger QikProp, LogP_app_ and *P*_app_ = experimental diffusion coefficient determined from a ratio-based method given as mean ± standard deviation (10^–6^ cm/s), LogP = determined from standard curve to measure [D] and [A], % diff (diffusion) = [acceptor]/[acceptor + donor], and recovery = (acceptor + donor)/total analyte, increase in % diffusion = difference between percent diffusion of macrocycle vs linear counterpart.

b Linear analog 2.4 = Boc deprotected.

**Figure 4:**
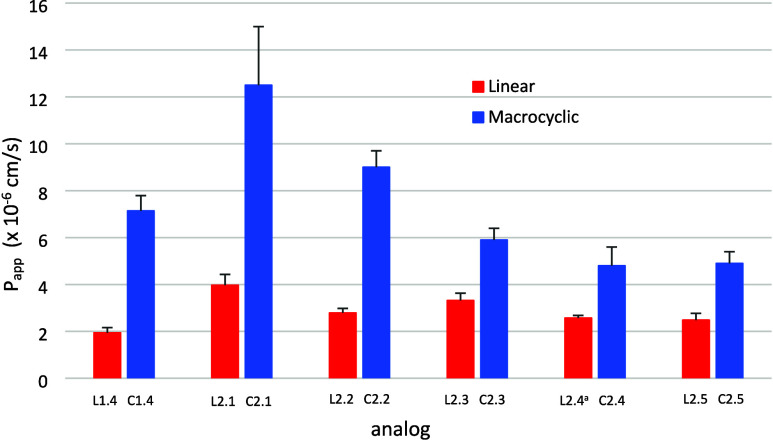
Effect of macrocyclization on passive membrane permeability: linear analogs (red) and macrocyclic analogs (blue). Values shown are the mean ± standard deviation. Definitions: *P*_app_ = rate of diffusion across artificial membrane (cm/s), analog = name of analog in series 2. ^*a*^Compound L2.4 is Boc-deprotected (see SI).

This study continues our interest in the use of oligomeric depsipeptides as a model for studying structure–permeability relationships in support of hit-to-lead studies. Classification of *ent-*verticilide (C1.4) as a bRo5 molecule derives from its 24-membered cyclic oligomeric depsipeptide structure with a molecular weight of 853 Da. Its oligomeric feature provides an opportunity to systematically modify its four repeating didepsipeptide units, with alternating lipophilic side chains (methyl, pentyl) and a polar backbone consisting of alternating esters and *N*-Me amides. End-to-end cyclization does not disrupt the character of the side chains, thereby allowing their contributions to remain constant across analogs. This contrasts with the common tactic of using side chains of two peptide residues to create a ring.

Cyclosporine A (CSA) is a prominent and well-studied example of a 33-membered cyclic peptide featuring passive transcellular absorption, metabolic stability, and oral administration.^22–24^ An exploratory pharmacokinetic study based on CSA revealed that backbone cyclization can act as a key strategy for modulating its cell permeability^23^ On the basis of a cell-based permeability assay with RRCK cells (Ralph Russ canine kidney), the cyclic form of CSA provided a nearly 10-fold increase in the rate of diffusion (*P*_app_ = 5.6 × 10^–6^ compared to 0.6 × 10^–6^ cm/s).

Hoffman^14,25^ discovered a trend in permeability while optimizing the pharmacokinetic profile of lead compound BL3020, a cyclic peptide melanocortin agonist. The studies featured 5 and 12 analogs, respectively, 4 of which were protected linear analogs. The modifications focused on side chain, ring sizes (20–23), and peptide-to-peptoid substitutions. Collectively, these analogs showed that 13 cyclic peptides exhibited up to 50–60% higher permeability than the 5 linear forms (Caco-2 permeability assay). They also noted the metabolic instability of the linear derivatives.

James and co-workers^26^ utilized two-solvent partitioning and an NMR study of diffusion coefficients for pairs of linear and cyclic molecules across a diverse range of molecular weights. Throughout a series of 8 analogs, ranging from 300 to 730 Da, they found an overall decrease in the rate of diffusion as size increased. They also showed that there is a statistically significant increase in solution diffusion following cyclization. Polyfunctional macrocycles (ring sizes 12–29) were formed via a copper catalyzed azide–alkyne cycloaddition (CuAAC), featuring a triazole, as well as 1–3 amide bonds, aryl groups, and ethers. An acyclic to cyclic trend tracks without any surprises, suggesting changes in diffusion are not responsible for the aberrant behavior of some macrocycles.

Pei and co-workers^27^ revealed that peptide bicyclization through disulfide bonds can increase both proteolytic stability and cell permeability. Upon camouflaging of the active linear precursor in a bicyclic ring system, the molecule gains access to an intracellular target and then undergoes disulfide bond reduction to reveal the active linear form. The enhancement of cellular uptake with the cyclic peptide illustrates an example where cyclization improved the pharmacokinetic properties.

Conversely, Kodadek and co-workers^28^ reported a comparison of 15-membered cyclic peptide steroid conjugates with their linear precursors using a reporter gene-based assay in HeLa cells to measure permeability. The study comprised 7 side chain modified stapled peptides with molecular weights around 600 Da. It was shown that the macrocyclic forms are not more permeable than their linear counterparts and the enhancement of permeability is negligible.

Two questions were posed for this study. First, we wondered whether the cyclic feature is a critical determinant of permeability and whether the COD size imparts favorable permeability characteristics for the specific backbone structure. We further wondered whether cyclization within a single chain length while maintaining the backbone identity is a consistent permeability-enhancing tactic. While cyclization of a linear peptide is accepted as a tactic that can improve drug-like properties, very few studies have shown a clear and definable trend connecting size to passive permeability. These reports document an increase in permeability following cyclization but are constrained by the substrate scope and molecular weight range.

In series 1, we predicted that each didepsipeptide addition would decrease passive permeability as measured by *P*_app_ and % diffusion.^14^ According to Lipinski’s guidelines and literature precedence, the analogs with smaller molecular sizes (C1.1 and C1.2) should have higher diffusion rates compared to larger oligomers (C1.5 and C1.6).^29^ In cases where cyclization increases passive diffusion, the cause is uncertain. One hypothesis for this observation is the reduction in effective size of the peptide or a change in effective lipophilicity.

An uncertain variable to this study is the use of C- and N-terminal protecting groups to mask carboxylic acid and amine functionality for the linear analogs. It was noted above that the choice of terminal N- and C-protection (Boc and Bn, respectively) adds both mass and varying polarity effects as a necessary consequence of identifying a suitable linear form for each macrocycle. In the case of increasing oligomer sizes, the mass contribution decreases as a percentage of the total mass. Series 2, however, controls for this contribution by virtue of its consistent chain length. Since the series 2 linear series exhibited rather small permeability changes, we speculate that the larger permeability changes in series 1 linear analogs are due to other effects, such as conformational mobility and exposed polar surface area.

The linear analogs in series 1 yielded more predictable permeability data in comparison to the cyclic forms. Although there is an overall decrease in the passive diffusion with an increase in size (Figure 5), there is a narrow range of *P*_app_ (×10^–6^ cm/s) values for L1.1 (4.3 ± 0.6) through L1.5 (1.81 ± 0.2). A critical finding within series 1 is that the potential for favorable depsipeptide permeability can be size-dependent. As shown in Figure 5, 2 of the 6 compounds exhibited significantly higher permeabilities (1.1 and 1.4) when compared to the other oligomers (1.2, 1.3, 1.5, and 1.6). This indicates that the effect of backbone cyclization may be more nuanced than anticipated, but that various ring sizes should be considered in structure–permeability studies.

**Figure 5:**
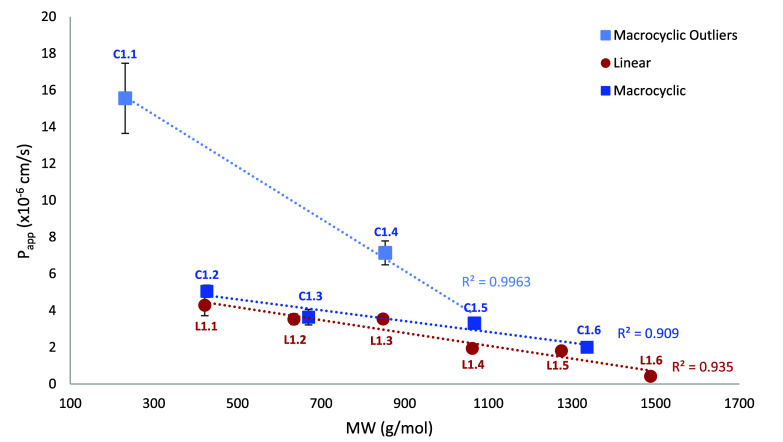
Comparison of molecular size to PAMPA passive membrane diffusion for series 1. Linear analogs are in red (in order: L1.1, 1.2, 1.3, 1.4, 1.5, 1.6), macrocyclic analogs are in blue (in order: C1.1, 1.2, 1.3, 1.4, 1.5, 1.6). Linear trend lines are shown with *R* ^2^ values. Definitions: *P*_app_ = rate of diffusion across artificial membrane (cm/s); MW = molecular weight (g/mol).

In parallel to series 1, the linear analogs in series 2 exhibited very similar permeabilities. There were minimal changes with each substitution of ester → N–H amide, with experimental *P*_app_ values ranging from 2.48 × 10^–6^ to 3.97 × 10^–6^ cm/s. The cyclization of two analogs (2.1 and 2.2) unearthed a more favorable orientation, possibly due to a conformational change induced via intramolecular H-bonding, capable of diffusing through the artificial membrane more rapidly than *ent*-verticilide by burying more polar groups to provide a lipophilic exterior.^15^ This further supports the finding that end-to-end backbone cyclization can lead to a significant increase in passive diffusion. The unusually large increases in permeability at ring sizes of 6 and 24 are more difficult to explain rigorously since other characteristics may contribute to permeability-driving behavior, including backbone relative stereochemistry, macrocycle conformational mobility, and the presentation of hydrogen bond acceptors. These factors will contribute to the rate of transfer through the artificial membrane, but the deconvolution of their roles is beyond the scope of this study.

A critical limitation in the field of bRo5 drug development and peptide hit-to-lead studies is the inability to rationally design complex membrane-permeable peptides. Most studies detailing the introduction of rings for conformational restriction have not been rigorously comprehensive owing to the asymmetry and structural limitations of the hit compound. This study addresses this need for a more comprehensive study that highlights the direct impact of backbone cyclization on (depsi)­peptide permeability. The ability to hold constant orthogonal contributing features such as side chain lipophilicity is a distinguishing feature. A highlight of series 1 is the documentation of decreasing permeability with increasing size (231–1488 Da), uniform increase in permeability upon cyclization, and the unusual benefit that cyclization can confer at specific ring sizes (Figure 6). A highlight of series 2 is the finding that cyclization again confers improved permeability relative to linear precursors but a nonuniform benefit within this narrowly focused size range (Figure 6). We speculate that the introduction of an N–H amide hydrogen bond donor creates an opportunity for an intramolecular hydrogen bond donor–acceptor interaction that is enhanced by cyclization.

**Figure 6:**
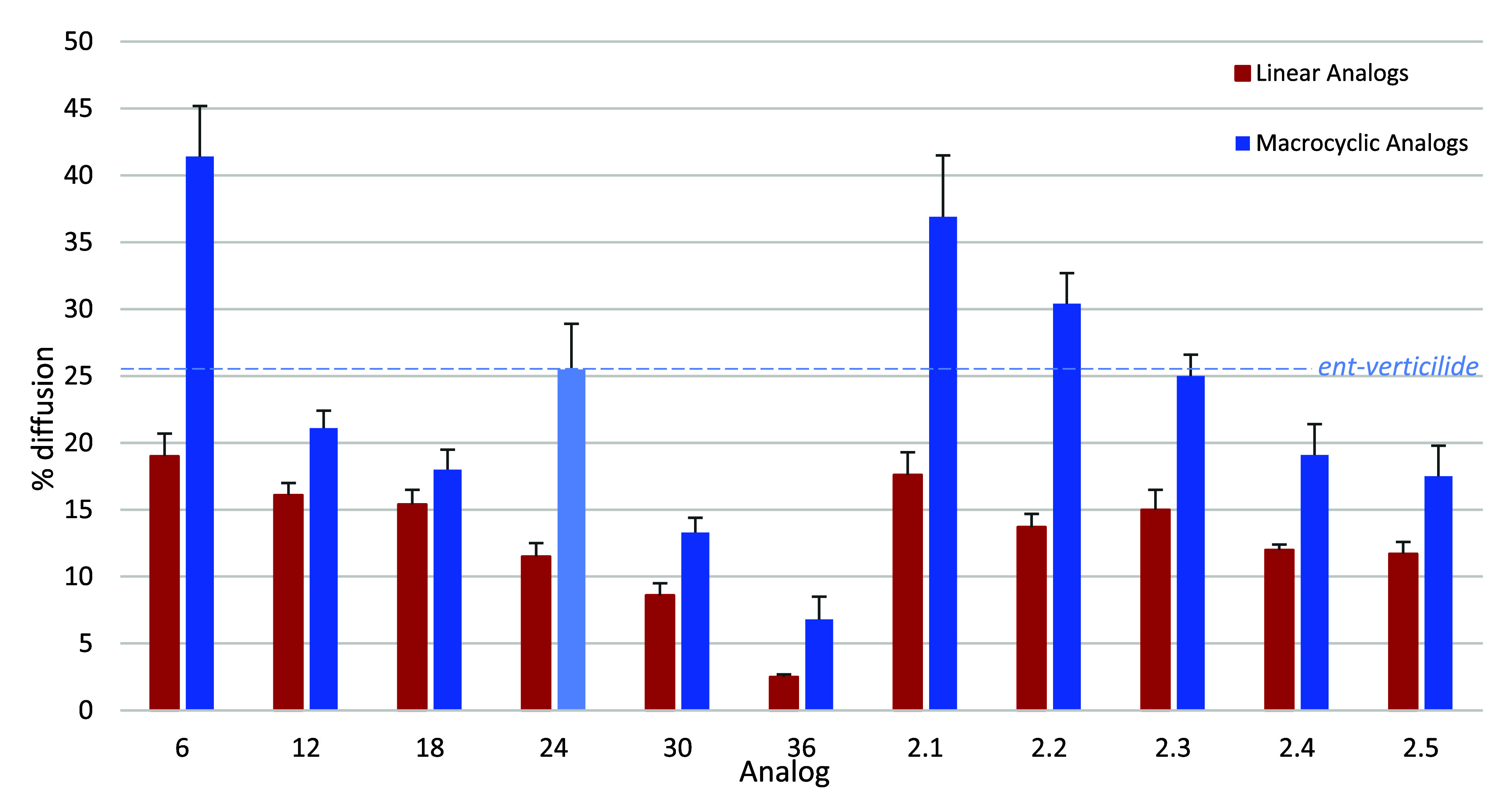
Overall summary of series 1 and 2 percent diffusion. Linear analogs for both series are in red, and macrocyclic analogs for both analogs are in blue. Error bars are ± the standard deviation. Lead compound *ent*-verticilide (24) is shown by a light blue line for comparison. Definitions: % diffusion = [acceptor]/[acceptor + donor].

## Safety

*No unexpected safety hazards were encountered during this work. For procedures described, standard precautions for synthetic organic chemistry were taken, including appropriate PPE. Some reaction solvents used to prepare compounds for this study are flammable and should therefore be kept away from sparks and other sources of ignition.*

## Materials and Methods

### Synthesis

Compounds were prepared by chemical synthesis and purified using silica gel chromatography to arrive at material that was >95% pure by ^1^H NMR spectroscopy and/or HPLC analysis, as described in previous publications.^15,30,31^

### PAMPA

The permeability assay across an artificial membrane was completed using literature procedures. Details are provided in the Supporting Information.^15,21^

## Supplementary Material





## Notes

Compound preparations, analytical data, spectroscopic data (PDF)
Molecular structures of all tested compounds, and associated data (CSV)

## Abbreviations

Ro5: Lipinski’s Rule-of-Five
COD: cyclic oligomeric depsipeptide
Papp: apparent permeability
HBD: hydrogen bond donor
PAMPA: Parallel Artificial Membrane Permeability Assay
L: linear
C: cyclic
MW: molecular weight (g/mol)
TPSA: topological polar surface area (Å)
QPLogP: calculated octanol/water logP using Schrödinger QikProp
*P*_app_: experimental diffusion coefficient determined from a ratio-based method given as mean ± standard deviation
LogP: determined from standard curve to measure [D] and [A]
% diff (diffusion): [acceptor]/[acceptor + donor]
recovery: (acceptor + donor)/total analyte
