# Supplementary material for: End-to-End Backbone Cyclization Enhances Passive Permeability of bRo5 Oligomeric Depsipeptides with Nonlinear Size Dependence
Source: ACS Med Chem Lett. 2025 Mar 20;16(4):638–45. doi: 10.1021/acsmedchemlett.5c00037 (PMC11995216; doi:10.1021/acsmedchemlett.5c00037)
Supplement: Supplementary file 1 [file ml5c00037_si_001.pdf]

End-to-End Backbone Cyclization Enhances Passive Permeability of bRo5 Oligomeric Depsipeptides with Nonlinear Size-Dependence

Madelaine P. Thorpe,[a] Corey R. Hopkins,[b] Jeffrey N. Johnston[a]*
[a]Department of Chemistry and Vanderbilt Institute of Chemical Biology, Vanderbilt University, Nashville, Tennessee 37235
[b]Department of Pharmaceutical Sciences, College of Pharmacy, University of Nebraska Medical Center, Omaha, NE 68198

*Corresponding Author: jeffrey.n.johnston@vanderbilt.edu

Table of Contents

I. Experimental Section ***2***

1.1 General Information and Procedures **2**
a. General Information 2
b. Purity Statement 2
c. General Procedures 3

1.2 PAMPA Experimental Procedure **3**

1.3 Materials and Suppliers **4**

II. Compounds ***5***

2.1 Macrocyclic Analogs **5**
a. Series 1: Ring-Size Variants 5
b. Series 2: Backbone Composition Variants 5

2.2 Linear Analogs **6**
a. Series 1: Ring-Size Variants 6
b. Series 2: Backbone Composition Variants 6

III. PAMPA Data ***7***

3.1 Control Experiments **7**
a. Positive Control: Propranolol Variables from Calculations and Standard Curve 7
b. Positive Control: Cyclosporine A Variables from Calculations and Standard Curve 8

3.2 Calculations **9**
a. Ratio Method Calculations ($LogP_{app}$ and P_{app}) 9
b. Standard Curve Method Calculations (LogP) 9
c. Percent Recovery (%R) 9
d. Percent Diffusion (% diff) 9

3.3 PAMPA Results **10**
a. *ent*-Verticilide: PAMPA Data and Standard Curves 10
b. Series 1 Macrocycles: PAMPA Data and Standard Curves 12
c. Series 1 Linear Analogs: PAMPA Data and Standard Curves 20
d. Series 2 Macrocyclic Analogs: PAMPA Data and Standard Curves 25
e. Series 2 Linear Analogs: PAMPA Data and Standard Curves 25

IV. Statistical Analysis ***29***

4.1 Series 1: Ring-Size Analogs **29**

a. Macrocyclic Variants ...29
b. Linear Variants ...30

4.2 Series 2: Backbone Composition Analogs ... 30
a. Macrocyclic Variants ...30
b. Linear Variants ...30

V. Calculated Data – Schrodinger QikProp ... 31

5.1 Comparison of Experimental Caco-2 Papp to Calculated LogP ... 32

VI. LC/MS and ELSD Traces ... 32

6.1 Macrocyclic Series ... 32
a. Series 1 ...32
b. Series 2 ...33

6.2 Linear Series ... 34
a. Series 1 ...34
b. Series 2 ...35

I. Experimental Section

1.1 General Information and Procedures

a. General Information

Glassware was flame-dried under vacuum for all non-aqueous reactions. All reagents and solvents were commercial grade and purified prior to use when necessary. Toluene, THF and dichloromethane (CH2Cl2) were dried by passage through a column of activated alumina as described by Grubbs.[1] Flash column chromatography was performed using Sorbent Technologies 230-400 mesh silica gel with solvent systems indicated. Analytical thin layer column chromatography was performed using Sorbent Technologies 250 µm glass backed UV254 silica gel plates and were visualized by fluorescence upon 250 nm radiation and/or the by use of TLC stain. Solvent removal was affected by rotary evaporation under vacuum (~ 25-40 mm Hg). All extracts were dried with Na2SO4 unless otherwise noted. Preparative HPLC was performed on an Agilent 1260 system (column: Zorbax Eclipse XDB-C18; 21.2 mm x 150 mm, 5 µm, flow rate 8 mL/min) with 210 nm monitoring wavelength and acetonitrile/water (+0.1% TFA) gradient as indicated. Nuclear magnetic resonance spectra (NMR) were acquired on a Bruker AV-400 (400 MHz) or Bruker AV II-600 (600 MHz) instrument. Mass spectra were recorded by use of electron impact ionization (EI), or electro-spray ionization (ESI) on a high resolution TQ-Orbitrap 3 XL Penn or Orbitrap 2 Classic FPG in the Vanderbilt Mass Spectrometry Core Laboratory. IR spectra were recorded on a Nicolet Avatar 360 spectrophotometer and are reported in wavenumbers (cm^{-1}) as neat films on a NaCl plate (transmission). Melting points were measured using an OptiMelt automated melting point system (Stanford Research Systems) and are not corrected. Chiral HPLC analysis was conducted on an Agilent 1200 series Infinity instrument using a ChiralPak column. Optical rotations were measured on a Jasco P-2000 polarimeter.

b. Purity Statement: Although each macrocycle was purified by preparative HPLC, and detected by UV (210 nm), the compounds reported lack a good chromophore. However, the use of HPLC in combination with NMR analysis led us to judge compounds to be a minimum of 90% pure, likely >95%. HPLC traces of final compounds are provided.

c. General Procedures:

***tert*-Butyloxycarbonyl (Boc) Deprotection**: A round-bottom flask was charged with the depsipeptide (1 equiv) and dissolved in either 4 M HCl/ethyl acetate (1 M in depsipeptide) or 20% TFA in DCM. The reaction was allowed to stir for 1-3 h at ambient temperature. The crude reaction mixture was concentrated, ether was added, and the mixture was then reconcentrated. This procedure was repeated 3 times with diethyl ether.

Benzyl Deprotection: A round-bottom flask was charged with the depsipeptide (1 equiv) dissolved in methanol or ethyl acetate (0.1 M) and treated with 10% Pd/C (20 mol%). The reaction flask was evacuated with a light vacuum (~40 Torr). Hydrogen (balloon) was added and then the flask was cycled through a light vacuum three times. The reaction was stirred for 1.5-3 h. The crude reaction mixture was filtered through Celite and concentrated to afford the carboxylic acid.

PyBrop Coupling Reaction: A round-bottom flask was charged with the two depsipeptide coupling partners (1:1), along with dry DCM or DMF (0.1 M). The solution was cooled to 0 °C and placed under argon. PyBrop (1.5 equiv) and freshly distilled DIPEA (3 equiv) were added. The reaction was allowed to stir for 30 minutes at 0 °C and 1.5-18 h at ambient temperature. Reaction progression was monitored by TLC and/or TLC-MS. The reaction mixture was poured into cold 10% aq citric acid and extracted with EtOAc. The combined organic layers were washed with satd aq $NaHCO_3$, brine, dried, and concentrated. The crude residue was subjected to flash column chromatography.

PyBOP Macrocyclization: The reaction mixture was added to a flame-dried round bottom flask. Dry DMF or DCM (0.005 M) was added and the reaction was cooled to 0 °C. Once at 0 °C, freshly distilled DIPEA (3 equiv) and PyBop (1.05 equiv) were added. The reaction was stirred at 0 °C for 30 minutes, then allowed to warm to ambient temperature and stir for an additional 1.5-3 h. Reaction progression was monitored by TLC and/or TLC-MS. The reaction mixture was poured into cold 10% aq citric acid and extracted with EtOAc. The combined organic layers were washed with satd aq $NaHCO_3$, brine, and then dried and concentrated. The crude reaction mixture was purified via preparative HPLC.

All analogs were prepared using previously reported protocols and spectral data are in agreement with literature values.[2,3,4]

1.2 PAMPA Experimental Procedure

PAMPA was conducted using a 96-well donor plate with 0.45 µm hydrophobic Immobilon-P membrane supports (Millipore), and a 96-well Teflon acceptor plate. 1000 µM stock solutions were made up of 1-4 mg of compound (dependent on molecular weight) and 100% DMSO. The donor solutions were prepared using 1.25 mL stock, 1.25 mL PBS (pH 7.4 – prepared as a 1L solution, Millipore P3813) and 80.0 mg D-α-Tocopherol polyethylene glycol 1000 succinate (TPGS, Millipore 57668). The donor concentration is 500 µm due to the limit of detection on the LC/MS system. The donor solutions were prepared using 38.4 mg TPGS and 5% DMSO in PBS (192 µL DMSO, 3684 µL PBS). A solution of 1% (w/v) of lecithin in *n*-dodecane was prepared, which acts to activate the membrane. Prior to setting up the experiments, each membrane

was visually assessed to ensure no cracking has occurred, which would skew the experiments. It is important to load the wells carefully to ensure the membranes don't become punctured. It is worth noting that once solutions are added working quickly is recommended to prevent evaporation. 5 µL of the lecithin solution was carefully applied to the membrane supports in the wells of the donor plate. The membrane should begin white and after application should be more transparent. The acceptor plate was prepared by adding 300 µL of the acceptor solution to each well. The donor plate was then placed on top of the acceptor plate and the acceptor solution should touch the membrane. It is important to begin recording times, to the second, as the donor solutions are added. Without allowing evaporation, 150 µL of the donor solution was added to each well. Once complete, the lid was placed on top of the donor plate, and the system was placed in a chamber with wet paper towels to prevent evaporation. It was incubated at room temperature for 14-18 hours and exact times were recorded to the second.

Once complete, the solutions were prepared to run on the LC/MS. 1-mL glass mass spectroscopy vials were labeled and the solutions were added to them. Rather than taking an aliquot of solution from each well, the entire solution was pulled up (either 150 µL or 300 µL) and placed in the vial. Then, the relative concentrations were analyzed by LC/MS performed on an Agilent 6130 single quadrupole LC-MS system. Chromatographic separation was performed on an Agilent Eclipse XD-C18 column (4.6x150mm, 5 µm) with 0.1% (vol) TFA in deionized water (mobile phase A) and 0.1% (vol) TFA in acetonitrile (mobile phase B). The following elution gradient was used: 30% B for 0.5 min, 30-75% linear gradient for 0.5 min, 75-88% linear gradient for 1 min, 88-90% linear gradient for 8 min, 90-100% linear gradient for 3 min, 100-30% linear gradient for 2 min. The flow rate was 1 mL/min. The column temperature was 23 °C and the injection volume was 100 µL. The ion source parameters of the mass spectrometer include gas temperature of 300 °C, gas flow of 12 L/min, nebulizer gas pressure of 30 psi, and sheath gas temperature of 200 °C. All peak-picking and integration bounds were manually entered for each spectrum.

1.3 Materials and Suppliers

- *n*-Dodecane: Oakwood Chemical – 099221
- Phosphate buffered saline pH 7.4 powder (PBS): Millipore – P-3813
- MultiScreen-IP PAMPA assay plates: Millipore – MAIPNTR10
- PTFE Acceptor plates: Millipore – MATRNPS50 or MSSACCEPT0R
- L-∂-phosphatidylcholine, egg lecithin: Sigma – P3556
- D-*α*-tocopheryl polyethylene glycol 1000 succinate (TPGS): Millipore – 57668
- Polysorbate 80 (TWEEN 80): Sigma – 1547969

II. Compounds

2.1 Macrocyclic Analogs

a. Series 1: Ring-Size Variants (C1.1-1.6)

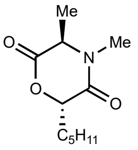

C1.1

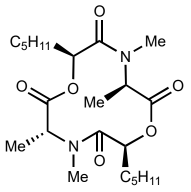

C1.2

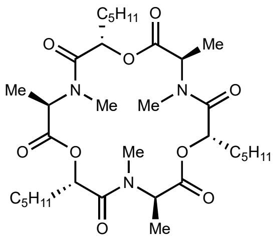

C1.3

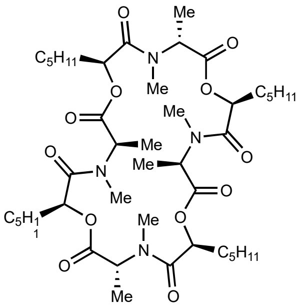

C1.4
ent-verticilide

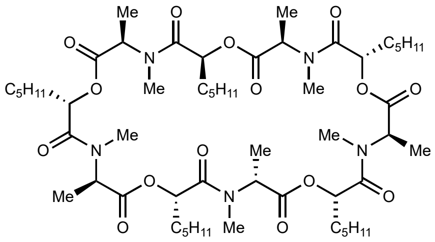

C1.5

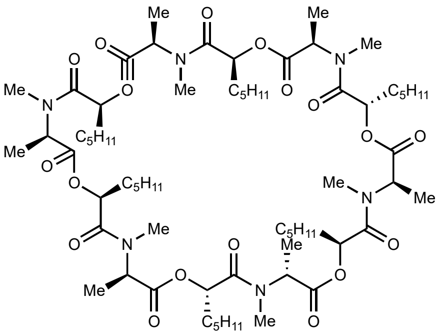

C1.6

b. Series 2: Backbone Composition Variants (C2.1-2.5)

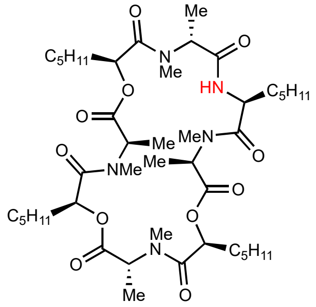

C2.1

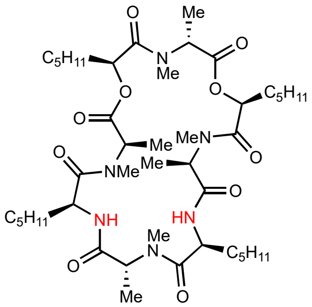

C2.2

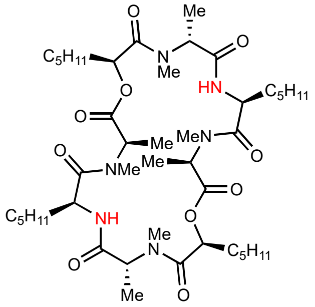

C2.3

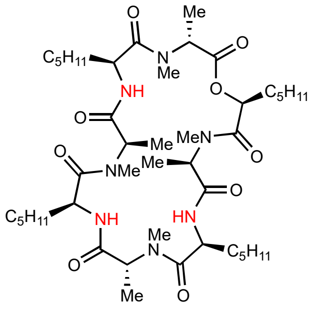

C2.4

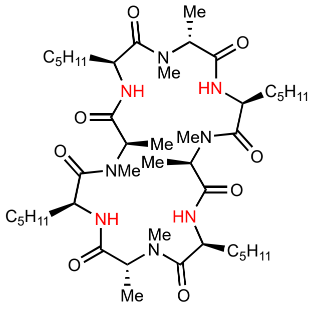

C2.5

2.2 Linear Analogs

a. Series 1: Ring-Size Variants (L1.1-1.6)

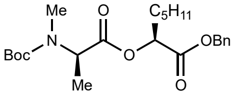

L1.1

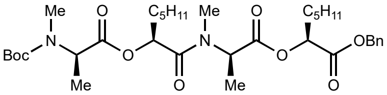

L1.2

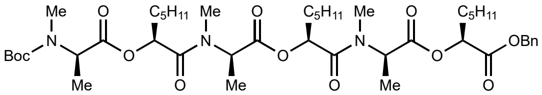

L1.3

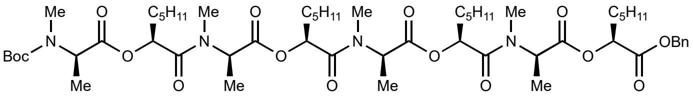

L1.4

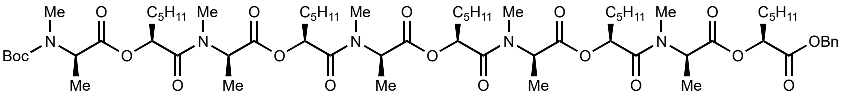

L1.5

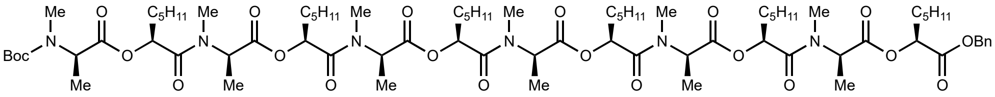

L1.6

b. Series 2: Backbone Composition Variants (L2.1-2.5)

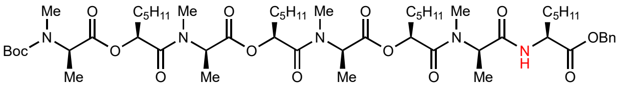

L2.1

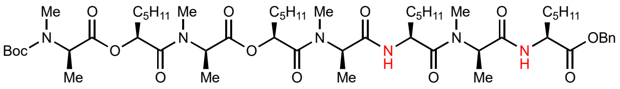

L2.2

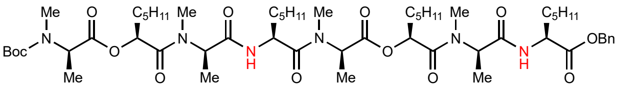

L2.3

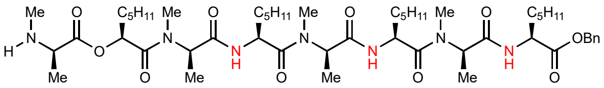

L2.4

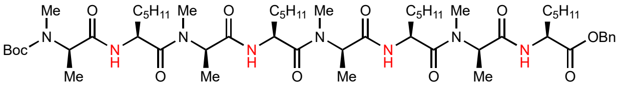

L2.5

III. PAMPA Data

3.1 Control Experiments

Prior to running the analogs in Series 1-2 of unknown permeability, rigorous optimization was completed. To ensure proper technique and reproducibility two standards of different molecular weights were subjected to the PAMPA conditions: propranolol and cyclosporin A. Literature values for these two positive controls were reproduced successfully.

a. Positive Control: Propranolol Variables from Calculations and Standard Curve

(*S*)-Propranolol (Millipore, catalog number P-0884) was subjected to standard PAMPA conditions listed above. Data was quantified using a UV trace, 254 nm. Each experiment included 5 replicates at a specified concentration (1: 10 uM, 2: 50 uM, 3: 100 uM, 4: 200 uM). The PAMPA data is shown below:

Exp	Ave. P_{app} (x 10^{-6})	Literature $P_{app}{}^{a}$ (x 10^{-6})	Well 1		Well 2		Well 3		Well 4		Well 5		C_0 (uM)	% diff. ± SD
			[acc]	[donor]	[acc]	[donor]	[acc]	[donor]	[acc]	[donor]	[acc]	[donor]		
1	9.4 ± 0.6	10.0 ± 0.0	4.05	5.06	3.97	5.05	3.95	4.94	4.03	4.97	3.92	5.13	10	37.1 ± 0.02
2	9.1 ± 0.4		18.26	28.51	17.46	29.06	18.31	29.83	18.07	31.26	18.09	29.62	50	
3	9.7 ± 0.4		37.89	55.45	33.45	57.74	36.13	58.01	35.56	54.55	39.92	58.91	100	
4	10.0 ± 0.3		72.80	113.27	71.05	118.47	73.31	115.52	71.12	116.80	71.60	112.16	200	

Supplement Table 1. Final Experimental Data for (S)-Propranolol. *The average calculated P_{app} was 9.55 ± 0.4 cm/s. The acceptor and donor well concentrations were calculated using a standard curve. The initial concentrations are listed under C_0. The average % diffusion is 37.1% ± 0.02. [a]Evaluation of the reproducibility of Parallel Artificial Membrane Permeation Assays (PAMPA). Schmidt, D., Lynch, J. 2003, Millipore. Definitions: [acc] = acceptor concentration, [donor] = donor concentration, % diff = % diffusion.*

Experiment	MW (g/mol)	Ave RT (min)	C (x10^{-9})	Well 1		Well 2		Well 3		Well 4		Well 5		Ave %R ± SD
				%T	%R	%T	%R	%T	%R	%T	%R	%T	%R	
1	259.34	3.85	3.23	0.81	91	0.80	90	0.81	89	0.83	90	0.77	90	93.1 ± 2.8
2			3.29	0.77	94	0.76	93	0.80	96	0.76	99	0.78	95	
3			3.04	0.55	93	0.60	91	0.59	94	0.59	90	0.61	99	
4			3.24	0.70	93	0.76	95	0.74	94	0.74	94	0.75	92	

Supplement Table 2. Variables from Calculations for (S)-Propranolol. *Data is summarized for 4 experiments with 5 replicates (20 total). The molecular weight is 259.34 g/mol and with a retention time of 3.9 minutes. Variable C and %T were calculated using Supplement Equation 1. The average % recovery was 93%. Definitions: MW = molecular weight, RT = retention time, %R = % recovery, Ave %R = average ± standard deviation.*

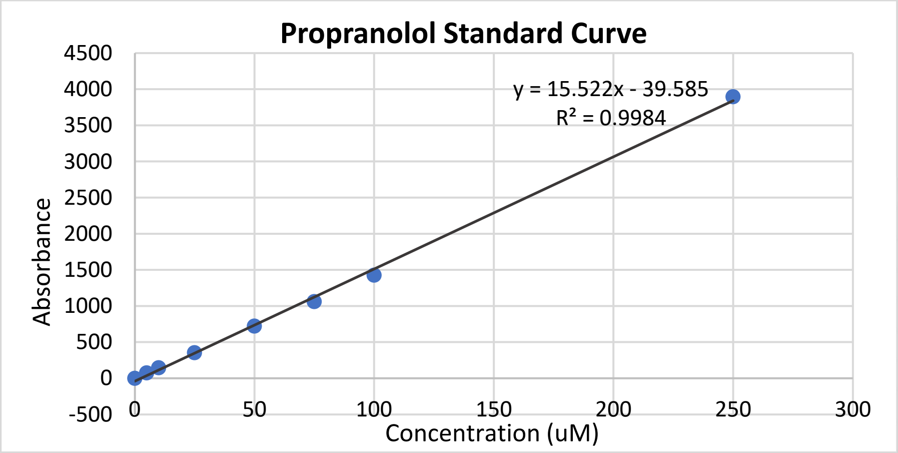

Conc. (uM)	Absorbance
250	3895.32
100	1426.7
75	1059.5
50	720.9
25	353.6
10	145.6
5	75.3
0	0

Supplement Graph 1. *The standard curve for absorbance vs. concentration (uM) of (S)-propranolol utilizing UV detection (254 nm).*

b. Positive Control: Cyclosporine A Variables from Calculations and Standard Curve

Cyclosporine A (Sigma Aldrich, catalog number C1832-5MG) was subjected to standard PAMPA conditions listed above. Data was quantified using a UV trace, 210 nm. Each experiment included 5 replicates at a concentration of 500 µM. This higher concentration was chosen following running samples at 50, 100, 250 µM and seeing weak peak absorbance. The limit of detection is 25 µM. The PAMPA data is shown below:

Experiment	MW (g/mol)	Ave RT (min)	C ($x10^{-9}$)	Well 1		Well 2		Well 3		Well 4		Well 5		Ave %R ± SD
				%T	%R	%T	%R	%T	%R	%T	%R	%T	%R	
1	1202.6	12.03	3.20	0.70	92	0.71	100	0.69	93	0.69	94	0.70	91	88.4 ± 5.9
2			3.11	0.65	97	0.65	89	0.67	92	0.67	90	0.65	86	
3			3.03	0.78	78	0.81	83	0.78	82	0.80	82	0.81	81	
4			3.21	0.74	84	0.74	92	0.77	82	0.72	93	0.74	87	

Supplement Table 3. Variables from Calculations for Cyclosporine A. *Data is summarized for 4 experiments with 5 replicates (20 total). The molecular weight is 1202.6 g/mol with a retention time of 12.03 minutes. Variable C and %T were calculated using Supplement Equation 1. The average % recovery was 88%. Definitions: MW = molecular weight, RT = retention time, %R = % recovery, Ave %R = average ± standard deviation.*

Exp	Ave. $LogP_{app}$ (x 10^{-6})	Literature $LogP_{app}$[a] (x 10^{-6})	Well 1		Well 2		Well 3		Well 4		Well 5		C_0 (uM)	% diff. ± SD
			[acc]	[donor]	[acc]	[donor]	[acc]	[donor]	[acc]	[donor]	[acc]	[donor]		
1	-5.06 ± 0.04	-5.01 ± 0.00	163.80	297.69	162.17	338.49	169.34	295.21	172.82	295.43	154.85	299.69	500	30.1 ± 0.02
2	-5.07 ± 0.03		163.89	321.67	149.84	293.96	154.98	306.98	160.44	291.70	151.52	277.27		
3	-5.08 ± 0.02		142.63	248.95	148.56	266.18	144.96	265.25	143.42	266.25	144.67	261.44		
4	-5.10 ± 0.02		135.17	285.46	146.79	312.02	138.79	270.43	141.78	321.87	139.48	293.97		

Supplement Table 4. Final Experimental Data for Cyclosporine A. *The average calculated $LogP_{app}$ was -5.07 ± 0.03 cm/s, calculated using Supplement Equation 1. The acceptor and donor well concentrations were calculated using a standard curve. The initial concentration is 500 uM. The average % diffusion is 30%. Definitions: [acc] = acceptor concentration, [donor] = donor concentration, % diff = % diffusion ± standard deviation.* [a]*Seo, J., et. al., J. Med. Chem.* ***2021****, 64, 8272.*

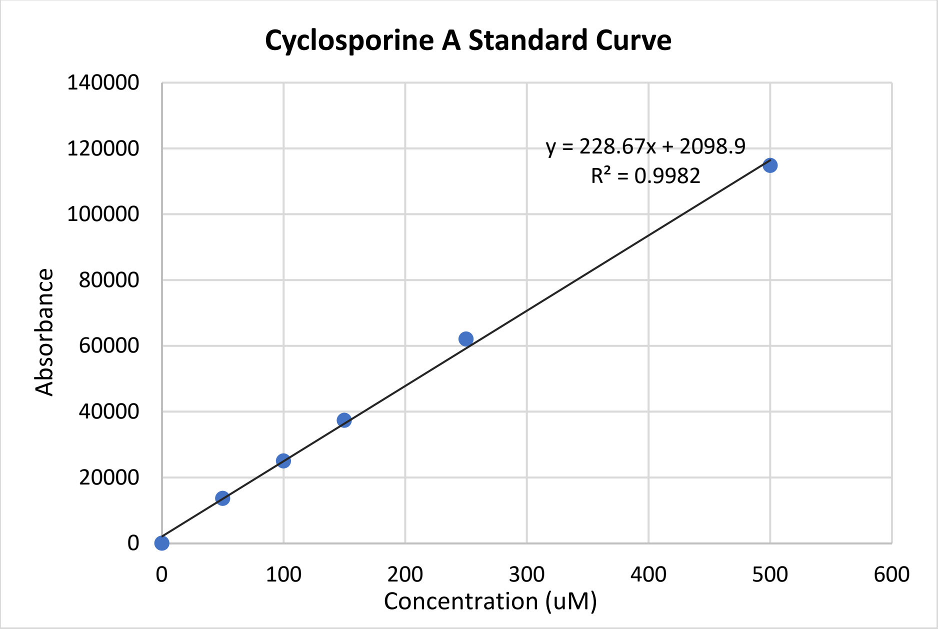

Conc. (uM)	Absorbance
500	114765.9
250	62031.8
150	37323.4
100	24980.1
50	13597.2
0	0

Supplement Graph 2. *The standard curve for absorbance vs. concentration (uM) of Cyclosporine A utilizing UV detection (210 nm).*

3.2 Calculations

a. Ratio Method Calculations (LogP$_{app}$ and P$_{app}$)

PAMPA permeability parameters were calculated by the following formulas:

$$C_{equilibrium} = \frac{(C_D V_D) + (C_A V_A)}{V_D + V_A}$$

$$\%T = \frac{C_A}{C_{equilibrium}} \times 100 = \frac{R_A}{\left(\frac{R_A V_A + R_D V_D}{V_A + V_D}\right)} \times 100$$

$$LogP_{app} = \log\left\{-\frac{V_D V_A}{V_D V_A} \times \frac{\ln(1\text{-}\%T)}{\text{area x time}}\right\}$$

$$\%\ Recovery = \frac{(C_D V_D + C_A V_A)}{C_0 V_D} \times 100$$

Supplement Equation 1. Equations for Calculating P_{app}. *Utilizing a ratio-based method of final concentrations (donor and acceptor wells), the following variables can be calculated - C_D: relative concentration in donor well, C_A: relative concentration in acceptor well, C_0: relative concentration of initial test solution added to donor well, V_D: volume of donor well (0.15 cm^3), V_A: volume of acceptor well (0.30 cm^3), Area: membrane area (0.24 cm^2), Time: actual elapsed time in second.*

b. Standard Curve Method Calculations (LogP)

Experimental LogP values were calculated by leveraging a standard curve to obtain concentrations in the donor and acceptor wells, then utilizing the following equation:

$$Log\ P = \log\left\{C \times \ln\left(1 - \frac{[analyte]_{acceptor}}{[analyte]_{donor}}\right)\right\} \quad \text{where } C = \frac{V_D \times V_A}{(V_D + V_A) \times \text{area x time}}$$

Supplement Equation 2. Equation for Calculating LogP. *LogP utilizes a standard curve-based method, where V_D = donor solution initial volume (300 uL) and V_A = acceptor solution initial volume (150 uL). LogP is the log of the effective permeability.*

c. Percent Recovery (%R)

Sample percent recovery was calculated using the following equation:

$$\text{Percent Recovery} = \frac{[acceptor] + [donor]}{500} \times 100$$

Supplement Equation 3. Percent Recovery Equation. *Total concentration of analyte detected after incubation divided by C_0.*

d. Percent Diffusion (% diff)

Percent diffusion is the amount of compound that diffused across the membrane:

$$\text{Percent Diffusion} = \frac{[acceptor]}{500} \times 100$$

Supplement Equation 4. Percent Diffusion Equation. *Percent diffusion is the [acceptor]/500 uM (C_0).*

3.3 PAMPA Results

a. *ent*-Verticilide: PAMPA Data and Standard Curves

Experiment	MW (g/mol)	Ave RT (min)	C ($x10^{-9}$)	Well 1		Well 2		Well 3		Well 4		Well 5		Ave %R
				%T	%R	%T	%R	%T	%R	%T	%R	%T	%R	
1			3.27	0.71	86	0.72	98	0.53	98	0.72	92	0.58	89	
2			3.20	0.66	99	0.62	94	0.72	88	0.67	96	0.75	86	
3			3.28	0.68	90	0.75	84	0.70	84	0.71	85	0.70	87	
4			3.27	0.74	88	0.71	87	0.72	84	0.53	93	0.72	93	
5	853.15	11.80	3.23	0.58	86	0.66	96	0.62	88	0.72	76	0.67	97	89.85 ± 5.64
6			3.28	0.75	86	0.67	83	0.75	86	0.70	87	0.71	77	
7			3.05	0.70	88	0.74	83	0.71	84	0.72	99	0.53	97	
8			3.20	0.72	96	0.58	99	0.66	97	0.62	95	0.72	85	
9			3.21	0.67	91	0.75	87	0.68	87	0.75	87	0.70	91	
10			3.24	0.71	91	0.70	88	0.74	93					

Supplement Table 5. The Variables from Calculations for ent-Verticilide. *ent-Verticilide was subjected to the optimized PAMPA conditions comprised of 6 experiments, each with 8 replicates run in tandem (total of 48 individual experiments). Dark grey boxes are unused. The molecular weight of ent-verticilide is 853.15 g/mol and the average retention time seen via HPLC (210 nm) is 10.1 minutes. The C and %T values are calculated using Supplement Equation* ***1****. The average percent recovery is 90%. Definitions: MW = molecular weight, RT = retention time, %R = % recovery, Ave %R = average ± standard deviation.*

Exp	Ave. $logP_{app}$	Ave. logP	Well 1		Well 2		Well 3		Well 4		Well 5		Ave. [acc]
			[acc]	[donor]	[acc]	[donor]	[acc]	[donor]	[acc]	[donor]	[acc]	[donor]	
1	-5.24	-9.00	137.13	294.39	156.71	332.05	109.93	380.22	150.20	310.31	139.99	305.40	
2	-5.13	-9.01	144.38	350.10	141.87	327.23	141.97	299.17	136.43	346.04	141.66	290.02	
3	-5.14	-8.99	128.70	322.90	136.90	283.18	123.79	295.27	128.35	294.44	129.00	307.89	
4	-5.14	-8.99	140.70	301.64	106.52	328.03	100.02	317.70	100.46	365.47	135.43	328.64	127.56 ± 17.17 uM (26%)
5	-5.37	-9.17	102.73	327.69	98.16	381.51	105.23	334.48	108.92	271.94	140.94	346.20	
6	-5.28	-9.21	141.38	289.87	94.39	323.10	144.66	284.12	141.48	295.46	91.72	294.34	
7	-5.26	-9.01	130.11	307.71	110.98	301.95	113.63	308.73	144.25	350.73	130.30	354.17	
8	-5.20	-9.03	133.07	348.96	119.98	375.87	159.32	323.79	151.74	321.96	115.39	360.24	
9	-5.19	-9.02	122.26	332.46	123.39	312.41	121.37	314.63	143.42	313.52	125.44	329.94	
10	-5.20	-9.00	147.93	304.65	125.61	312.06	124.96	337.91					

Supplement Table 6. Final Experimental Data for ent-Verticilide. *The average calculated $LogP_{app}$ is -5.22 ± 0.07 cm/s, calculated using Supplement Equation* ***1****. The average logP is -9.04 ± 0.08, calculated using Supplement Equation 2. The acceptor and donor well concentrations were calculated using a standard curve. The initial concentration is 500 uM for all experiments. The average acceptor concentration is 128 uM, yielding a percent diffusion of 26%. The dark grey boxes in the table are unused. There was a total of 48 experiments (6 individual experiments x 8 replicates run in tandem). Definitions: [acc] = acceptor concentration after incubation, [donor] = donor concentration after incubation, average [acc] = average acceptor concentration ± standard deviation.*

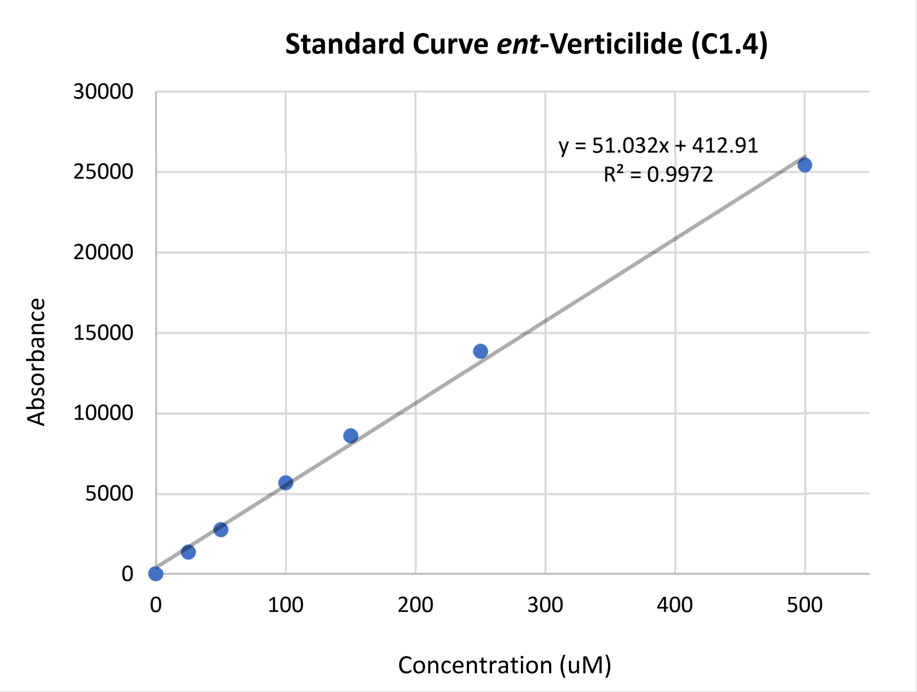

Conc. (uM)	Area
500	25422.4
250	13857.9
150	8604.07
100	5690.66
50	2784.89
25	1389.67
0	0

Supplement Graph 3. *The standard curve for absorbance vs. concentration (uM) of ent-verticilide utilizing* ***UV detection*** *(210 nm).*

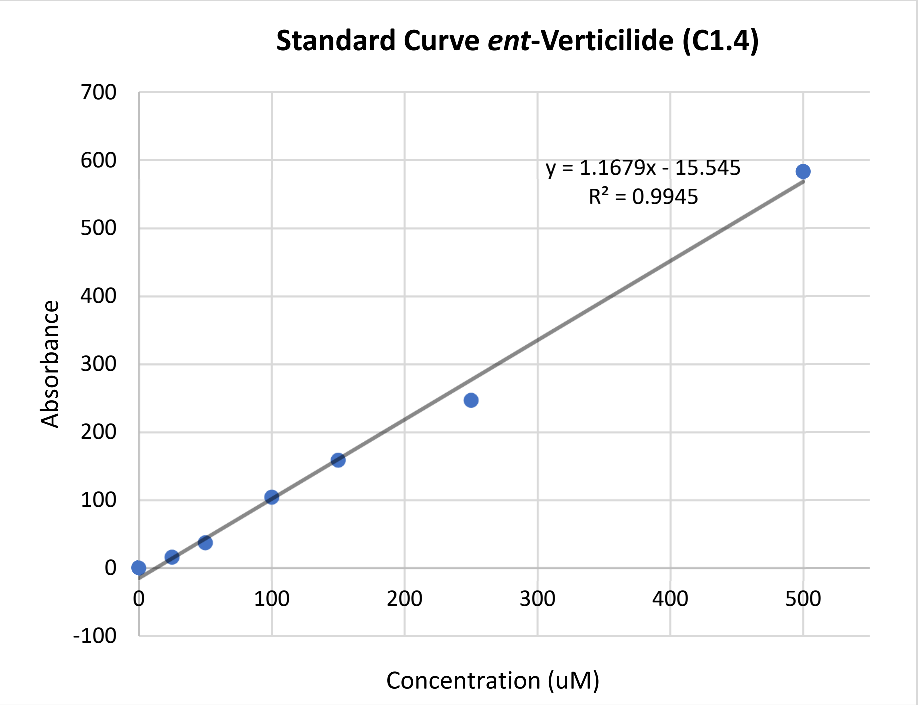

Conc. (uM)	Area
500	583.193
250	247.036
150	158.758
100	104.47
50	37.2842
25	15.9836
0	0

Supplement Graph 4. *The standard curve for absorbance vs. concentration (uM) of ent-verticilide utilizing* ***ELSD*** *detection.*

b. Series 1 Macrocycles: PAMPA Data and Standard Curves

Acceptor Concentration (uM) – UV Detection					
**C1.1**	**C1.2**	**C1.3**	**C1.4**	**C1.5**	**C1.6**
208.5	98.5	89.6	***see above section*** a. ent-Verticilide: PAMPA Data and Standard Curves	66.6	38.56
204.1	102.6	100.4		60.4	37.61
193.3	110.9	101.0		65.2	33.89
231.4	108.4	93.8		56.7	31.61
245.0	102.8	88.6		61.9	37.00
193.4	95.0	79.9		71.3	51.06
178.2	109.4	82.2		64.8	41.41
199.2	113.6	95.3		71.7	41.94
208.3	113.9	83.1		73.6	49.05
209.3	100.7	88.1		71.6	37.38
		84.0			
		81.4			
		95.3			
		92.2			
		85.4			
**Ave: 207.1**	**105.6**	**89.4**	**127.6**	**66.4**	**39.95**
**SD: 19.2**	**6.6**	**6.8**	**17.2**	**5.6**	**6.16**

Supplement Table 7. PAMPA acceptor concentrations (UV detection). *The acceptor concentrations were calculated using a standard curve. The initial concentration is 500 uM for all experiments. The average [acceptor] and standard deviations are listed in the final 2 rows of the table. The grey boxes in the table are unused. There were 10-24 experiments for each analog.*

Acceptor Concentration (uM) – ELSD Detection					
**C1.1**	**C1.2**	**C1.3**	**C1.4**	**C1.5**	**C1.6**
224.8	94.8	102.0	122.3	67.5	27.2
205.4	98.9	105.8	147.9	63.9	30.0
212.2	102.2	96.0	156.7	64.8	25.6
216.3	107.3	100.8	121.9	62.3	29.3
206.8	98.1	105.7	136.9	65.1	27.1
206.5	113.3	98.1	76.5	65.0	45.6
216.0	118.1	103.1	98.2	68.5	39.3
218.5	122.4	90.6	94.4	67.7	42.2
208.3	113.4	103.4	105.2	61.3	25.8
201.8	108.7	101.0	92.1	66.1	46.7
		96.3	113.6		
		106.6	159.3		
		111.5	121.4		
		102.0	125.0		
		94.9	150.2		
			136.4		
			141.7		
			129.0		
			90.2		
			59.8		
			91.7		
			130.3		
			115.4		
			125.4		
**Ave: 211.7**	**107.7**	**101.2**	**118.4**	**65.2**	**33.9**
**SD: 7.1**	**9.2**	**5.3**	**25.7**	**2.3**	**8.6**

Supplement Table 8. PAMPA acceptor concentrations (ELSD detection). *The acceptor concentrations were calculated using a standard curve. The initial concentration is 500 uM for all experiments. The average [acceptor] and standard deviations are listed in the final 2 rows of the table. The grey boxes in the table are unused. There were 10-24 experiments for each analog.*

Supplement Tables 9 and 10. PAMPA Papp Values: *The Papp were calculated using* ***Supplement Equation 1****. The average Papp and standard deviations are listed in the final 2 rows of the table. The top table is using UV detection and the bottom is using ELSD detection. The grey boxes in the tablze are unused. There were 10-24 experiments for each analog.*

**Papp (x 10^{-6} cm/s) – UV Detection**					
**C1.1**	**C1.2**	**C1.3**	**C1.4**	**C1.5**	**C1.6**
15.09	4.63	3.32	6.39	3.30	1.95
14.22	4.93	4.01	7.95	3.13	1.93
12.73	5.16	4.37	6.48	3.08	1.95
17.99	5.20	3.67	7.85	2.85	1.81
19.00	5.05	3.75	6.83	3.14	1.90
13.77	4.49	3.00	7.11	3.54	2.59
11.22	5.18	3.19	6.85	3.19	1.89
12.68	5.55	4.00	7.63	3.60	1.91
14.33	5.44	3.36	4.68	3.64	2.19
15.01	4.87	3.70	4.63	3.64	1.87
		3.24	4.05		
		3.17	6.98		
		3.78	4.62		
		3.81	3.79		
		3.57	4.66		
			5.98		
			6.44		
			7.86		
			4.86		
			6.27		
			6.77		
			5.18		
			5.55		
			6.05		
**Ave: 14.60**	**5.05**	**3.64**	**7.14**	**3.31**	**2.00**
**SD: 2.38**	**0.33**	**0.42**	**0.65**	**0.28**	**0.23**

**Papp (x 10^{-6} cm/s) – ELSD Detection**					
**C1.1**	**C1.2**	**C1.3**	**C1.4**	**C1.5**	**C1.6**
14.34	4.36	4.16	7.05	2.88	1.90
13.56	4.48	4.41	7.20	2.93	1.94
13.86	4.72	3.96	4.28	2.62	1.99
15.45	4.83	4.13	7.39	2.78	1.87
14.97	4.34	4.45	4.99	3.09	1.89
11.66	4.86	4.03	6.24	2.80	1.93
14.14	4.88	4.24	5.58	3.15	1.88
14.97	5.23	3.64	7.27	2.83	1.91
14.81	5.23	4.31	4.68	2.67	1.80
13.58	4.89	4.39	4.63	3.08	1.92
		3.96	4.05		
		4.40	6.98		
		5.05	4.62		
		4.44	3.79		
		4.01	4.66		
			5.98		
			5.46		
			6.2		
			5.51		
			5.72		
			4.75		
			7.56		
			7.22		
			4.78		
**Ave: 14.13**	**4.78**	**4.24**	**5.69**	**2.88**	**1.90**
**SD: 1.08**	**0.32**	**0.32**	**1.19**	**0.18**	**0.05**

Cmpd	MW (g/mol)	Ave RT (min)	C ($x10^{-9}$)	Well 1		Well 2		Well 3		Well 4		Well 5		Ave. %R
				%T	%R	%T	%R	%T	%R	%T	%R	%T	%R	
**C1.1**	231.3	12.0	2.99	0.91	93	0.86	93	0.89	92	0.92	99	0.93	99	94.00 ± 3.28
				0.93	89	0.92	90	0.89	95	0.96	95	0.99	94	
**C1.2**	426.6	11.4	3.11	0.56	91	0.58	90	0.60	93	0.60	91	0.59	88	90.70 ± 1.41
				0.55	90	0.60	92	0.62	90	0.61	92	0.57	89	
**C1.3**	669.8	13.1	3.13	0.44	97	0.51	94	0.54	88	0.48	94	0.48	87	91.24 ± 3.44
				0.41	94	0.43	92	0.51	89	0.45	89	0.48	87	
				0.44	93	0.43	91	0.49	94	0.48	90	0.47	87	
**C1.4**	853.1	11.8	3.22	***see above section*** a. ent-Verticilide: PAMPA Data and Standard Curves										89.85 ± 5.64
**C1.5**	1066.4	13.9	3.23	0.44	89	0.42	86	0.42	93	0.39	89	0.42	88	88.71 ± 1.96
				0.46	89	0.43	90	0.47	88	0.47	89	0.47	87	
**C1.6**	1335.8	14.3	3.20	0.63	89	0.65	88	0.63	80	0.66	82	0.62	89	89.74 ± 6.17
				0.58	86	0.63	97	0.59	98	0.60	97	0.63	91	

Supplement Table 11. The Variables from Calculations for Macrocyclic Analogs. *Analog ent-6 data is comprised of 2 experiments, each with 5 replicates run in tandem (10 total). Analog ent-12 data is comprised of 2 experiments, each with 5 replicates run in tandem (10 total). Analog ent-18 (ent-B1) data is comprised of 3 experiments, each with 5 replicates run in tandem (15 total). Analog ent-24 (ent-vert) data is comprised of 3 experiments, each with 8 replicates run in tandem (24 total). Analog ent-30 data is comprised of 2 experiments, each with 5 replicates run in tandem (10 total). Analog ent-36 data is comprised of 2 experiments, each with 5 replicates run in tandem (10 total). The molecular weights and mean retention times observed by HPLC (210 nm) are listed. The C values and %T values are calculated using Supplement Equation **1**. The mean percent recoveries are listed. The data shown is only for UV detection. Definitions: MW = molecular weight, RT = retention time, %R = % recovery, Ave. %R = mean ± standard deviation.*

Cmpd	Ave. $logP_{app}$	Ave. logP	Well 1		Well 2		Well 3		Well 4		Well 5		Ave. [acc]	Ave. % diff
			[acc]	[donor]	[acc]	[donor]	[acc]	[donor]	[acc]	[donor]	[acc]	[donor]		
**C1.1**	-4.82 ± 0.11	-8.76 ± 0.05	208.5	258.6	204.1	262.2	193.2	267.0	231.4	263.9	245.0	250.2	207.1 ± 19.2	41.42 ± 3.84
			193.4	253.4	178.2	270.7	199.2	275.9	208.3	266.4	209.3	260.6		
**C1.2**	-5.30 ± 0.03	-9.12 ± 0.03	98.5	356.3	102.6	346.8	110.9	355.8	108.4	345.5	102.8	339.3	105.6 ± 6.6	21.11 ± 1.31
			95.0	356.3	109.4	350.1	113.6	337.9	113.9	345.5	100.7	345.9		
**C1.3**	-5.44 ± 0.05	-9.19 ± 0.04	89.6	396.5	100.4	369.9	101.0	341.3	93.8	376.7	88.6	347.4	89.4 ± 6.8	18.04 ± 1.47
			79.9	389.6	82.2	376.5	95.3	350.9	83.1	362.2	88.1	349.1		
			84.0	380.0	81.4	374.5	95.3	372.2	92.2	356.5	85.4	351.0		
**C1.4**	-5.21 ± 0.09	-9.03 ± 0.07	***see above section*** a. ent-Verticilide: PAMPA Data and Standard Curves										127.56 ± 17.17	26%
**C1.5**	-5.48 ± 0.04	-9.34 ± 0.04	66.6	379.0	60.4	369.7	65.2	400.5	56.7	388.8	61.8	376.1	66.38 ± 5.63	13.28 ± 1.13
			71.3	372.2	64.8	384.1	71.7	367.3	73.6	371.8	71.6	362.1		
**C1.6**	-5.70 ± 0.04	-9.57 ± 0.07	38.6	407.4	37.6	403.5	33.9	368.2	31.6	379.4	37.0	405.9	33.88 ± 8.57	6.78 ± 1.71
			51.1	377.0	41.4	445.0	41.9	445.7	49.1	438.0	37.4	417.3		

Supplement Table 12. Final Experimental Data for Macrocyclic Analogs. *The mean $logP_{app}$ (calculated from Supplement Equation 1) and logP values (calculated from (Supplement Equation 2) are listed. The acceptor and donor concentrations are calculated using a standard curve and the units are uM. The initial concentration is 500 uM for all experiments. The total mean acceptor concentrations are listed in the final column.). The data shown is only for UV detection. Definitions: [acc] = acceptor concentration after incubation, [donor] = donor concentration after incubation, mean [acc] = mean acceptor concentration ± standard deviation.*

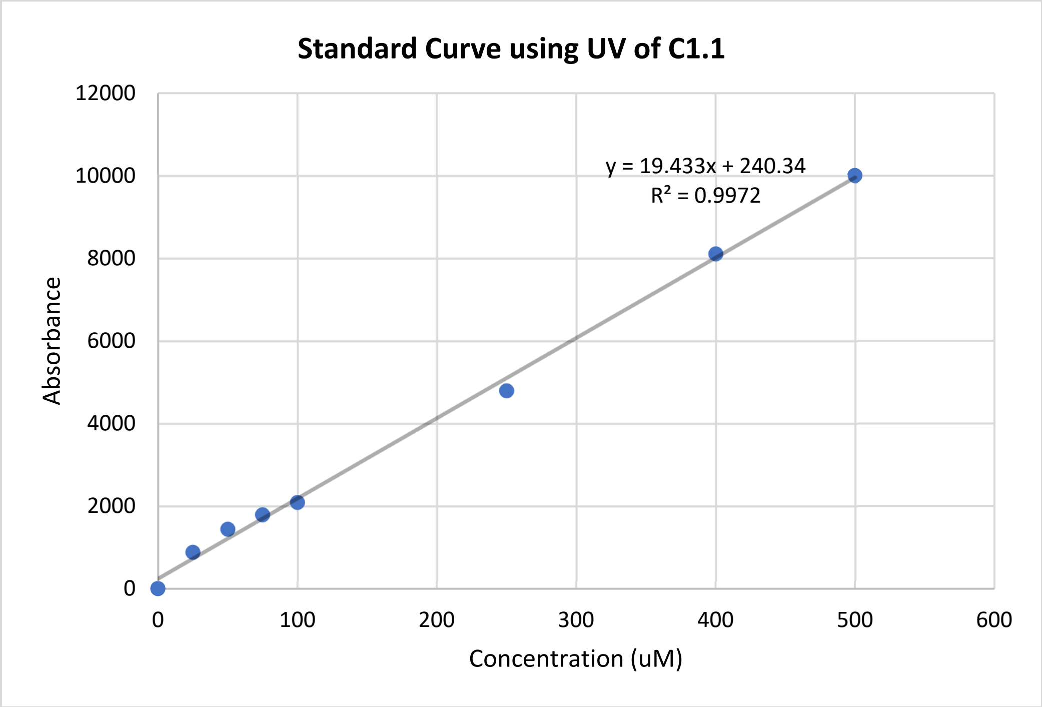

Conc. (uM)	Area
500	10003.1
400	8109.38
250	4796.88
100	2093.43
75	1793.66
50	1443.53
25	888.49
0	0

Supplement Graph 5. *The standard curve for absorbance vs. concentration of analog ent-6 utilizing* ***UV detection*** *(210 nm).*

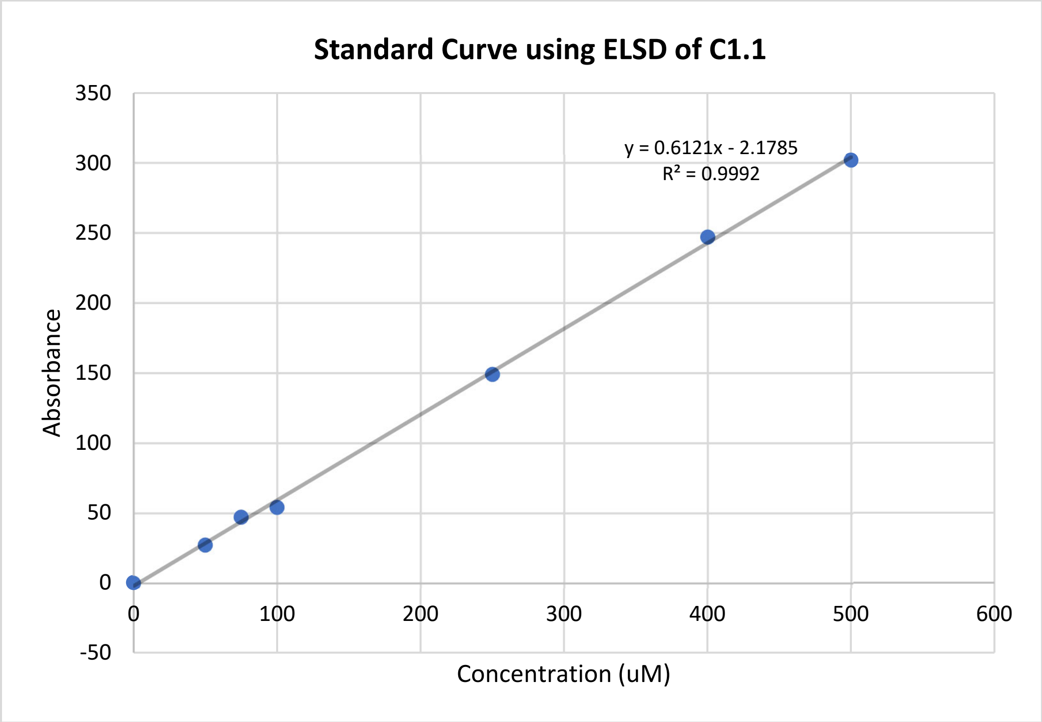

Conc. (uM)	Area
500	301.93
400	246.93
250	149.01
100	54.08
75	47.193
50	27.19
0	0

Supplement Graph 6. *The standard curve for absorbance vs. concentration (uM) of analog ent-6 utilizing* ***ELSD detection****.*

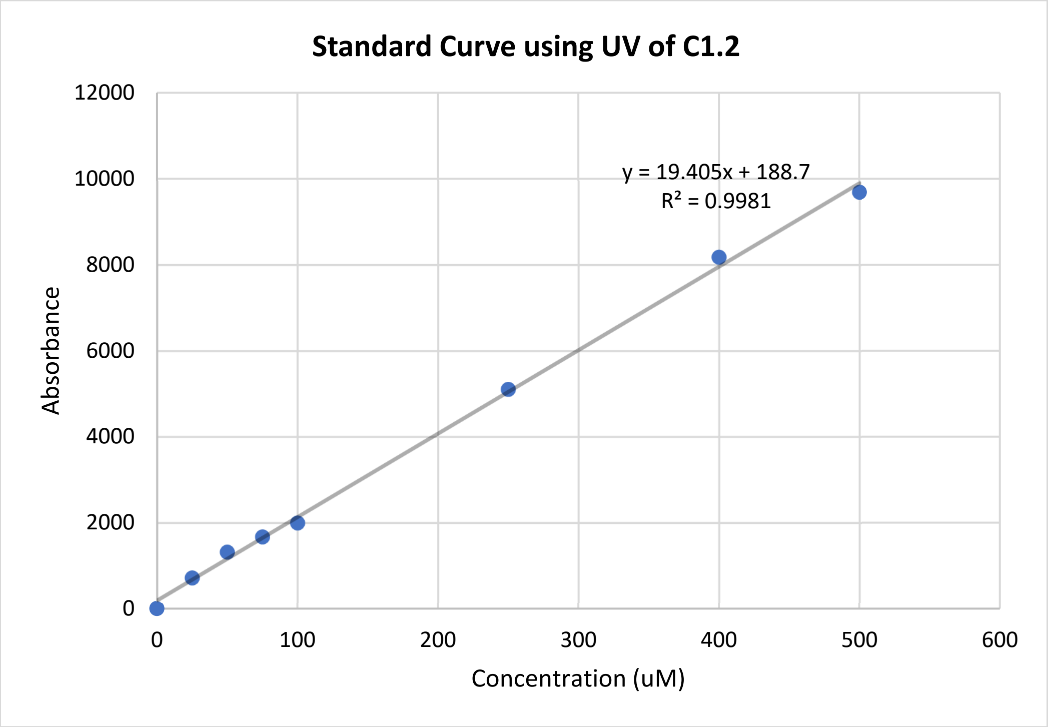

Conc. (uM)	Area
500	9684
400	8175
250	5102
100	1998
75	1675
50	1321
25	722
0	0

Supplement Graph 7. *The standard curve for absorbance vs. concentration of analog ent-12 utilizing* ***UV detection*** *(210 nm).*

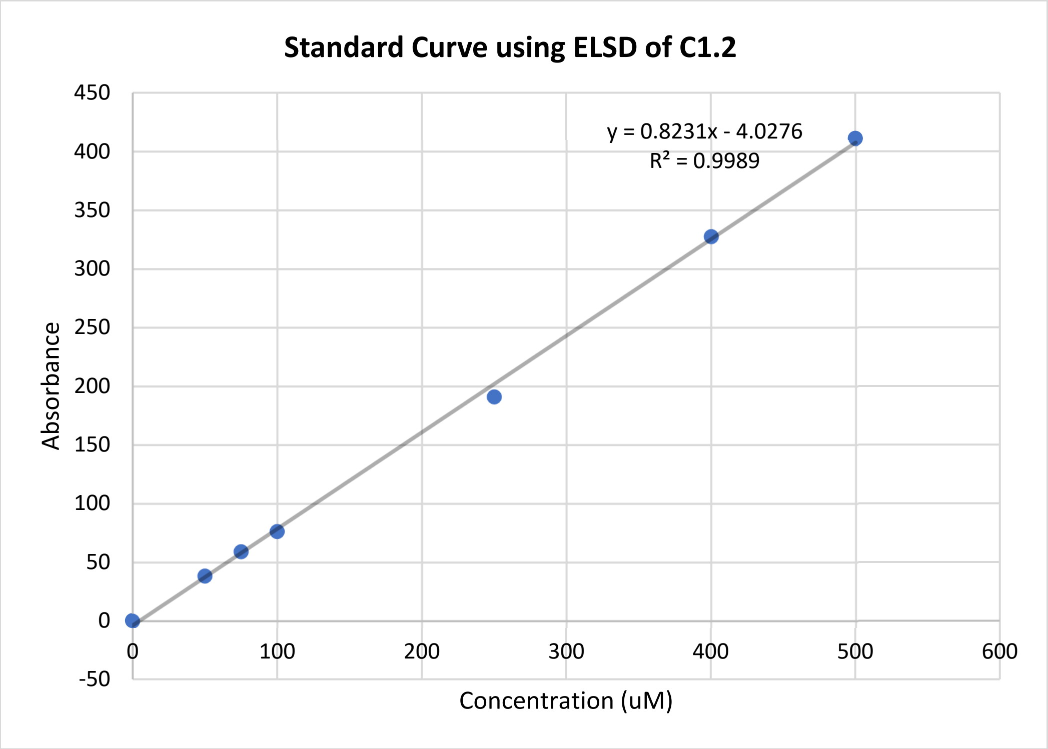

Conc. (uM)	Area
500	411
400	327.6
250	191
100	76.3
75	59.2
50	38.43
0	0

Supplement Graph 8. *The standard curve for absorbance vs. concentration (uM) of analog ent-12 utilizing* ***ELSD detection****.*

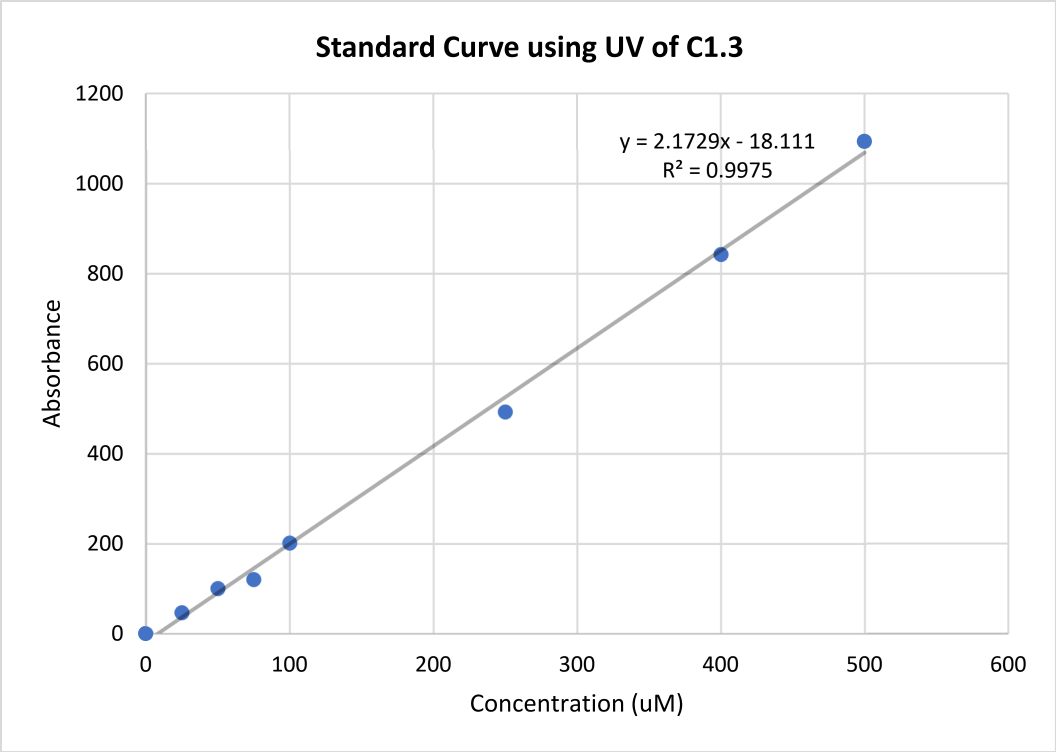

Conc. (uM)	Area
500	1093.32
400	842.3043
250	492.542
100	201.42
75	120.445
50	100.4757
25	46.667
0	0

Supplement Graph 9. *The standard curve for absorbance vs. concentration of analog ent-18 (B1) utilizing* ***UV detection*** *(210 nm).*

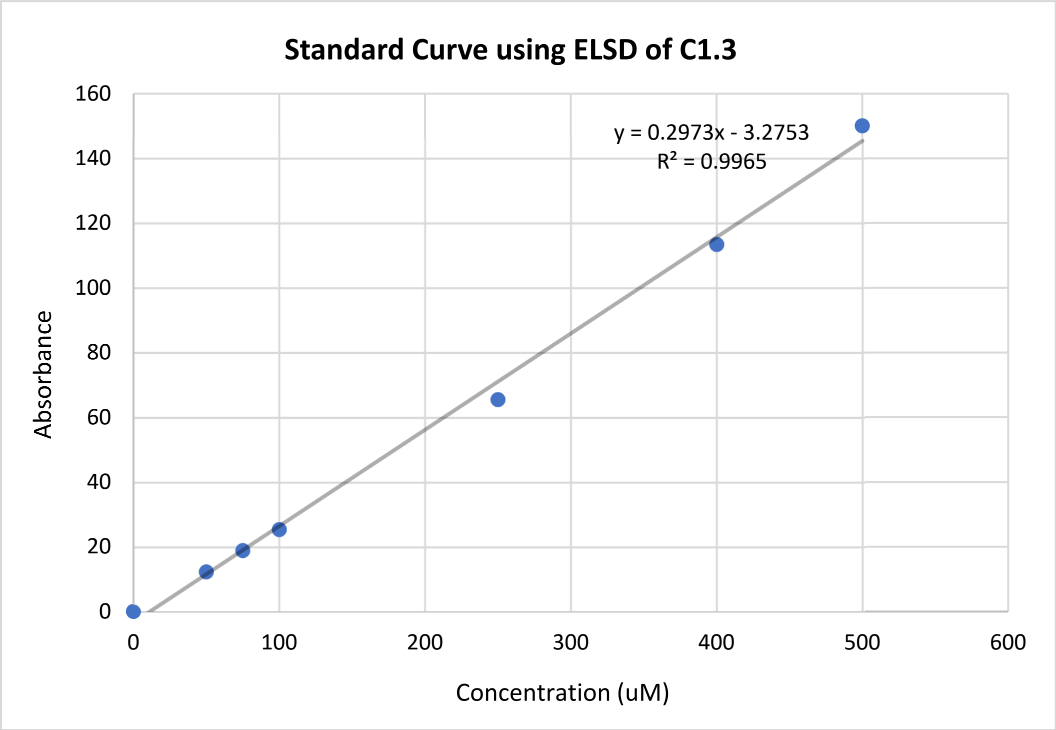

Conc. (uM)	Area
500	149.994
400	113.443
250	65.596
100	25.455
75	18.998
50	12.384
0	0

Supplement Graph 10. *The standard curve for absorbance vs. concentration (uM) of analog ent-18 (B1) utilizing* ***ELSD detection****.*

ent-24 (_ent_-vert): _see above section_ **a. ent-Verticilide: PAMPA Data and Standard Curves**

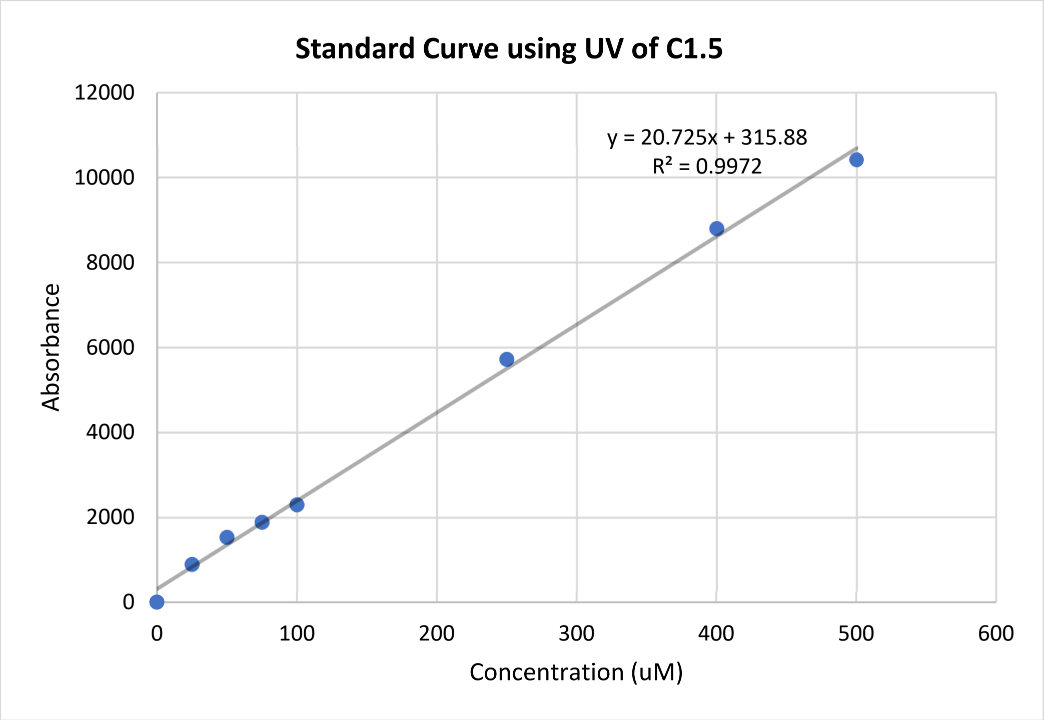

Conc. (uM)	Area
500	10411.7
400	8793
250	5719.4
100	2297.6
75	1888
50	1536
25	896.6
0	0

Supplement Graph 11. *The standard curve for absorbance vs. concentration of analog ent-30 utilizing* ***UV detection*** *(210 nm).*

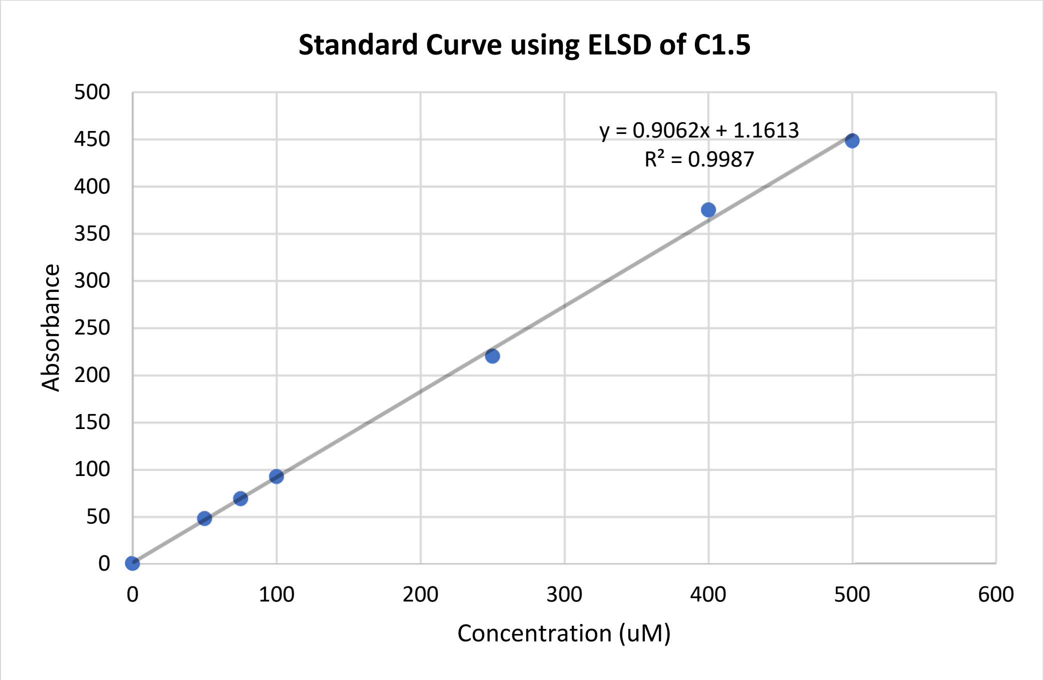

Conc. (uM)	Area
500	448.3
400	375.2
250	220.3
100	92.7
75	69.32
50	48.3
0	0

Supplement Graph 12. *The standard curve for absorbance vs. concentration (uM) of analog ent-30 utilizing* ***ELSD detection****.*

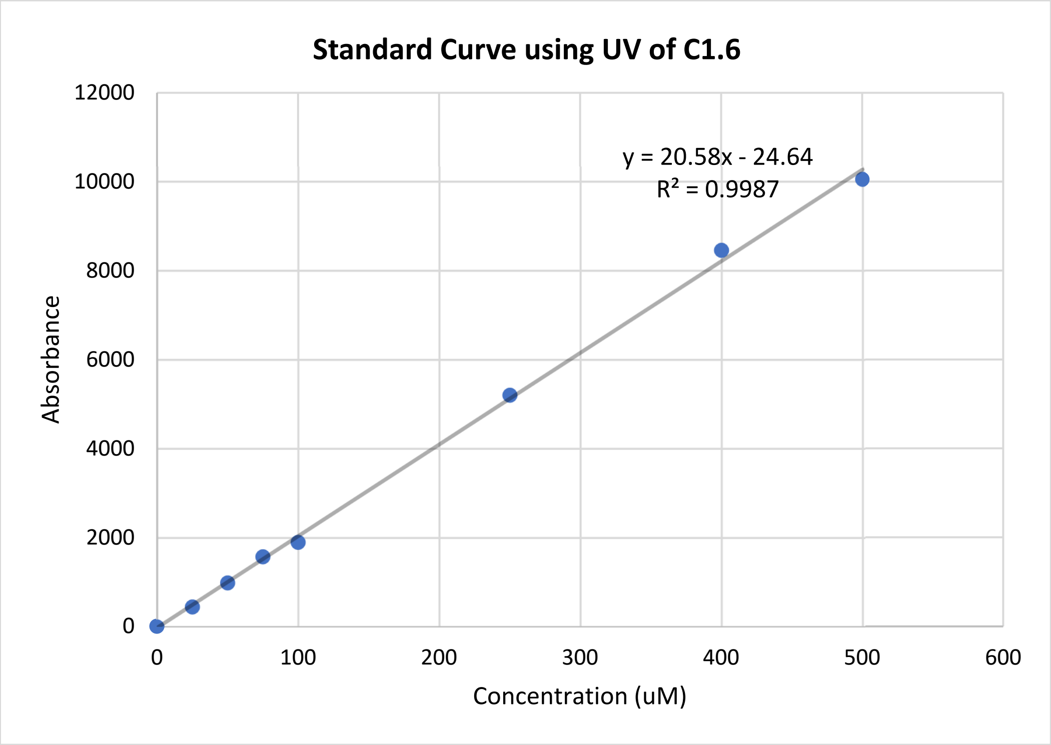

Conc. (uM)	Area
500	10049.01
400	8455.602
250	5201.35
100	1899.32
75	1573.12
50	988.67
25	448.42
0	0

Supplement Graph 13. *The standard curve for absorbance vs. concentration of analog ent-36 utilizing* ***UV detection*** *(210 nm).*

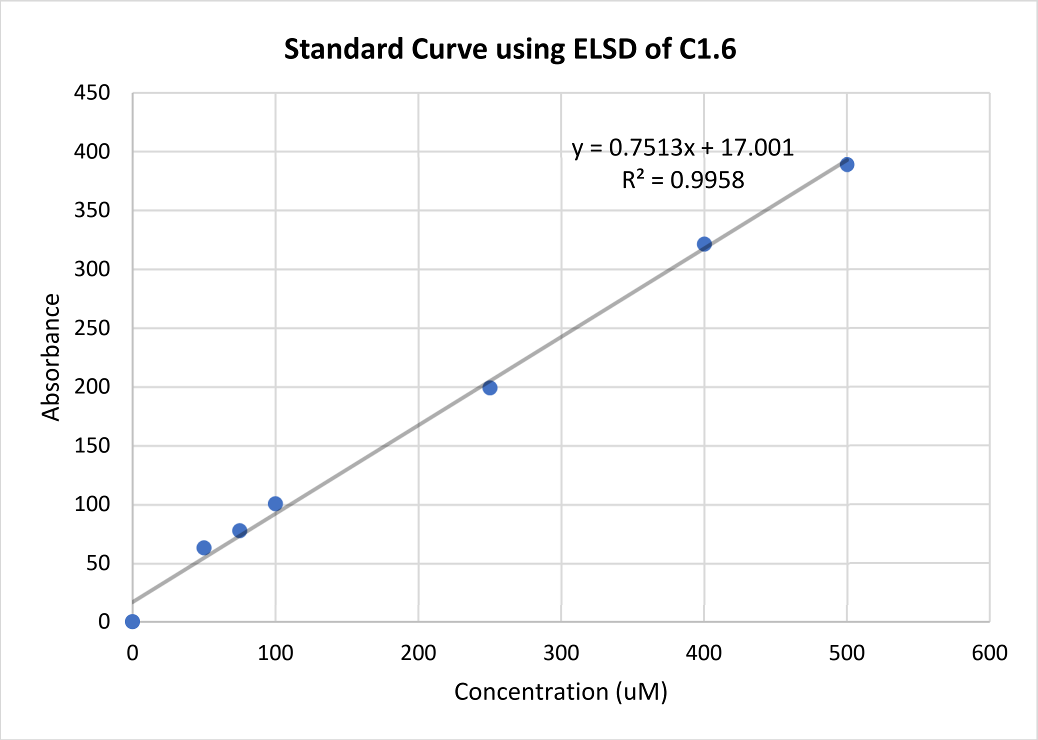

Conc. (uM)	Area
500	388.93
400	321.43
250	199.29
100	100.92
75	78.02
50	63.43
0	0

Supplement Graph 14. *The standard curve for absorbance vs. concentration (uM) of analog ent-30 utilizing* ***ELSD detection****.*

c. Series 1 Linear Analogs: PAMPA Data and Standard Curves

Acceptor Concentration (uM)					
L1.1	L1.2	L1.3	L1.4	L1.5	L1.6
97.9	72.0	84.1	52.3	50.2	***below limit of detection***
95.4	80.6	80.7	57.9	38.4	
79.7	77.4	74.5	64.4	40.2	
94.3	75.2	72.5	61.4	40.8	
84.7	77.1	68.1	62.2	37.5	
107.0	90.3	71.8	61.3	47.1	
95.8	86.9	84.0	51.5	40.3	
92.9	80.4	79.0	55.0	41.0	
106.7	87.3	81.4	59.1	45.3	
97.4	83.7	75.6	51.7	48.0	
**Ave: 95.2**	**78.0**	**77.2**	**57.7**	**42.9**	**< 25**
**SD: 8.4**	**3.2**	**5.5**	**4.7**	**4.4**	**NA**

Supplement Table 13. PAMPA acceptor concentrations (UV detection) for Linear Analogs. *The acceptor concentrations were calculated using a standard curve. The initial concentration is 500 uM for all experiments. The average [acceptor] and standard deviations are listed in the final 2 rows of the table. There were 10 experiments for each analog.*

Papp (x 10^{-6} cm/s)					
L1.1	L1.2	L1.3	L1.4	L1.5	L1.6
4.67	3.2	3.86	1.61	2.29	0.40
4.06	3.8	3.52	2.02	1.55	0.44
3.27	3.8	3.52	2.01	1.77	0.41
4.23	3.7	3.49	2.03	1.78	0.42
3.87	3.9	3.18	2.24	1.56	0.44
4.51	3.4	3.34	2.09	1.90	0.43
4.10	3.9	3.62	1.63	1.61	0.41
4.51	3.3	3.46	1.88	1.79	0.43
5.45	3.7	3.83	2.15	1.89	0.42
4.19	3.6	3.57	1.75	1.94	0.40
**Ave: 4.29**	**3.44**	**3.54**	**1.94**	**1.81**	**0.42**
**SD: 0.57**	**0.33**	**0.20**	**0.22**	**0.22**	**0.01**

Supplement Table 14. PAMPA Papp Values (UV detection) for Linear Analogs. *The Papp were calculated using* ***Supplement Equation 1****. The average Papp and standard deviations are listed in the final 2 rows of the table. There were 10 experiments for each analog.*

Cmpd	MW (g/mol)	Ave RT (min)	C (x10^{-9})	Well 1		Well 2		Well 3		Well 4		Well 5		Ave. %R
				%T	%R	%T	%R	%T	%R	%T	%R	%T	%R	
L1.1	421.5	11.2	3.03	0.56	87	0.51	96	0.44	97	0.52	91	0.49	89	91.90 ± 5.05
				0.55	98	0.51	95	0.55	86	0.62	84	0.52	95	
L1.2	634.8	13.6	3.21	0.43	93	0.48	88	0.48	85	0.48	84	0.50	82	88.24 ± 4.42
				0.47	88	0.46	96	0.47	90	0.49	85	0.49	88	
L1.3	848.1	11.8	3.19	0.44	86	0.47	93	0.46	91	0.49	86	0.47	85	87.71 ± 3.37
				0.49	89	0.46	92	0.46	85	0.46	84	0.43	86	
L1.4	1061.4	12.0	3.23	0.25	94	0.30	87	0.30	99	0.31	93	0.33	87	90.04 ± 4.46
				0.31	90	0.25	92	0.28	87	0.32	85	0.26	86	
L1.5	1274.6	9.3	3.22	0.33	83	0.24	93	0.27	85	0.27	86	0.24	90	89.40 ± 3.90
				0.28	93	0.25	94	0.27	86	0.29	90	0.29	93	
L1.6	1487.9	7.6	3.23	0.32	87	0.37	80	0.44	85	0.41	83	0.42	81	83.74 ± 2.76
				0.44	82	0.33	87	0.40	82	0.41	83	0.37	88	

Supplement Table 15. The Variables from Calculations for Linear Analogs. *Analog linear-6 data is comprised of 2 experiments, each with 5 replicates run in tandem (10 total). Analog linear-12 data is comprised of 2 experiments, each with 5 replicates run in tandem (10 total). Analog linear-18 data is comprised of 2 experiments, each with 5 replicates run in tandem (10 total). Analog linear-24 data is comprised of 2 experiments, each with 5 replicates run in tandem (10 total). Analog linear-30 data is comprised of 2 experiments, each with 5 replicates run in tandem (10 total). Analog linear-36 data is comprised of 2 experiments, each with 5 replicates run in tandem (10 total). The molecular weights and mean retention times seen via HPLC (210 nm) are listed. The C values and %T values are calculated using Supplement Equation* ***1***. *The mean percent recoveries are listed. The data shown is only for UV detection. Definitions: MW = molecular weight, RT = retention time, %R = % recovery, Ave. %R = mean ± standard deviation.*

Cmpd	Ave. $logP_{app}$	Ave. logP	Well 1		Well 2		Well 3		Well 4		Well 5		Ave. [acc]	Ave. % diff
			[acc]	[donor]	[acc]	[donor]	[acc]	[donor]	[acc]	[donor]	[acc]	[donor]		
L1.1	-5.37 ± 0.06	-9.17 ± 0.04	97.9	339.1	95.4	383.5	79.7	405.6	94.3	363.1	84.7	359.7	95.2 ± 8.4	19.03 ± 1.69
			107.0	383.0	95.8	380.6	92.9	335.0	106.7	314.8	97.4	378.9		
L1.2	-5.74 ± 0.04	-9.26 ± 0.02	72.0	391.3	80.6	361.1	77.4	348.2	75.2	344.8	77.1	331.0	78.01 ± 3.19	16.07 ± 0.88
			77.0	364.0	81.5	396.5	79.5	368.3	78.8	348.2	81.8	358.5		
L1.3	-5.45 ± 0.02	-9.27 ± 0.03	71.8	357.9	84	382.3	79.0	378.2	81.4	350.2	75.6	350.9	77.17 ± 5.49	15.43 ± 1.10
			84.1	358.3	80.7	379.4	74.5	351.2	72.5	345.9	68.1	359.8		
L1.4	-5.71 ± 0.05	-9.04 ± 0.04	52.3	417.0	57.9	378.9	64.4	431.9	61.4	403.7	62.2	372.2	57.69 ± 4.75	11.54 ± 0.95
			61.3	390.2	51.5	406.3	55.0	381.8	59.1	364.0	51.7	379.3		
L1.5	-5.75 ± 0.05	-9.54 ± 0.05	50.2	366.2	38.4	424.6	40.2	386.8	40.8	389.7	37.5	412.3	42.87 ± 4.41	8.57 ± 0.88
			47.1	418.9	40.3	429.3	41.0	389.2	45.3	405.5	48.0	418.7		
L1.6	-6.38 ± 0.01	ND	ND	435.3	ND	398.2	ND	424.4	ND	416.4	ND	403.2	ND	< 5%
			ND	410.5	ND	433.6	ND	409.7	ND	414.7	ND	437.5		

Supplement Table 16. Final Experimental Data for Macrocyclic Analogs. *The mean $logP_{app}$ (calculated from Supplement Equation 1) and logP values (calculated from (Supplement Equation 2) are listed. The acceptor and donor concentrations are calculated using a standard curve and the units are uM. The initial concentration is 500 uM for all experiments. The total mean acceptor concentrations are listed in the final column.). The data shown is only for UV detection. Definitions: [acc] = acceptor concentration after incubation, [donor] = donor concentration after incubation, mean [acc] = mean acceptor concentration ± standard deviation.*

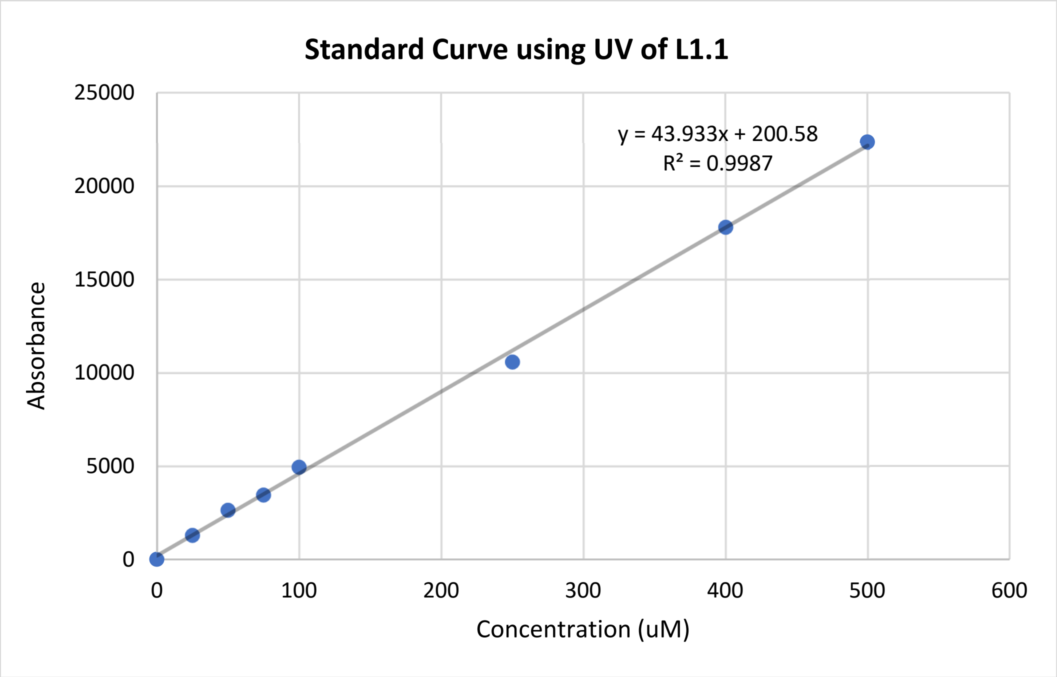

Conc. (uM)	Area
500	22350.4
400	17800.3
250	10588.7
100	4956.77
75	3465.16
50	2650.4
25	1299.6
0	0

Supplement Graph 15. *The standard curve for absorbance vs. concentration of analog Linear-6 utilizing* ***UV detection*** *(210 nm).*

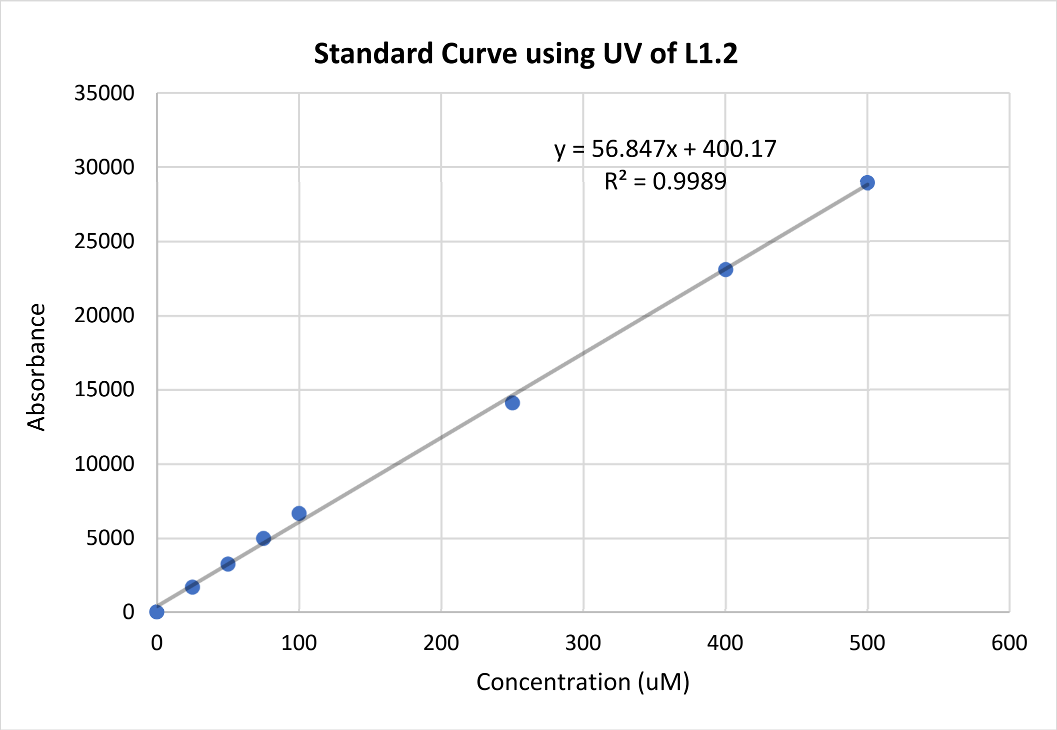

Conc. (uM)	Area
500	28938.5
400	23100.4
250	14119.1
100	6670.99
75	4991.5
50	3262.3
25	1704.5
0	0

Supplement Graph 16. *The standard curve for absorbance vs. concentration of analog Linear-12 utilizing* ***UV detection*** *(210 nm).*

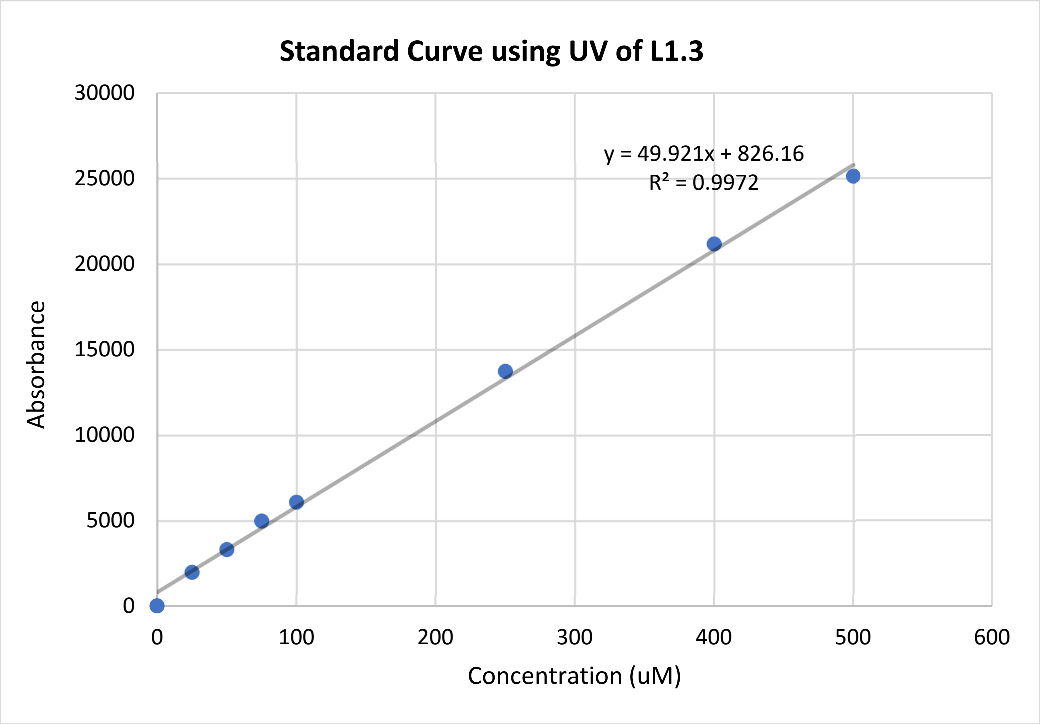

Conc. (uM)	Area
500	25140.98
400	21181.48
250	13742.23
100	6102.98
75	4999.51
50	3329.42
25	2002.4
0	0

Supplement Graph 17. *The standard curve for absorbance vs. concentration of analog Linear-18 utilizing* ***UV detection*** *(210 nm).*

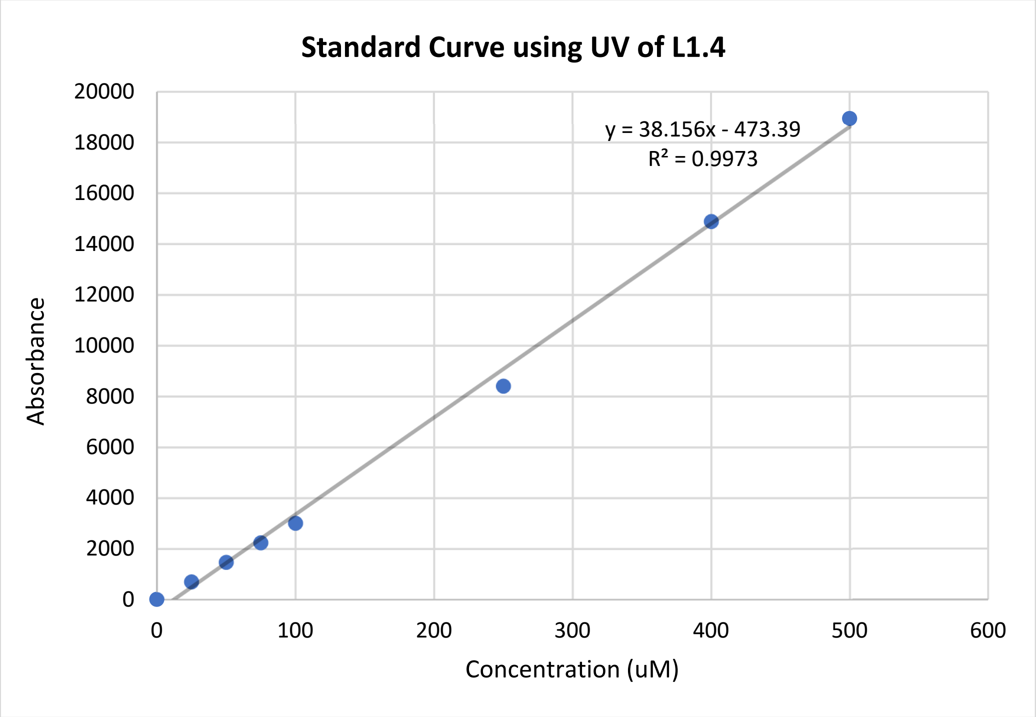

Conc. (uM)	Area
500	18937.5
400	14883.4
250	8398.83
100	3009.41
75	2235.11
50	1467.31
25	699.31
0	0

Supplement Graph 18. *The standard curve for absorbance vs. concentration of analog Linear-24 utilizing* ***UV detection*** *(210 nm).*

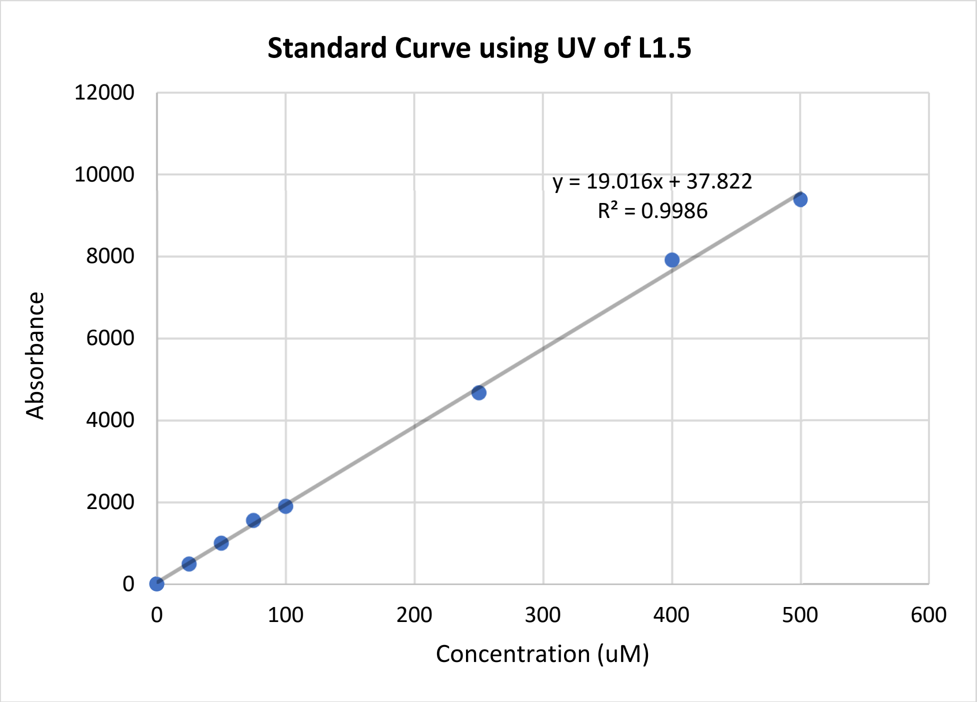

Conc. (uM)	Area
500	9384.1
400	7912.3
250	4672.94
100	1901.31
75	1556.43
50	1003.53
25	494.5
0	0

Supplement Graph 19. *The standard curve for absorbance vs. concentration of analog Linear-30 utilizing **UV detection** (210 nm).*

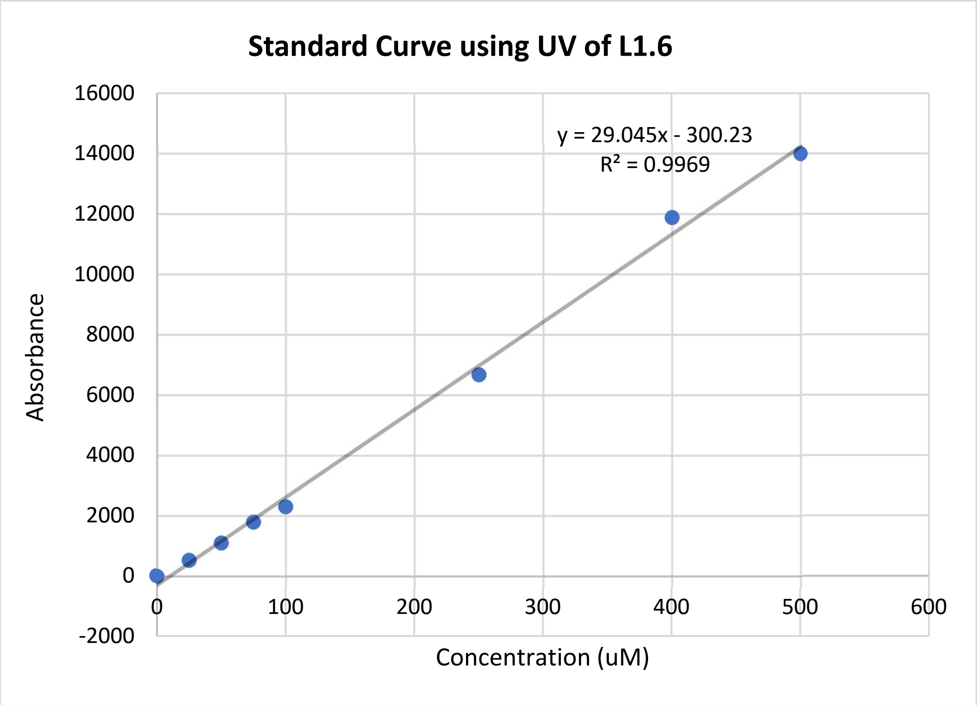

Conc. (uM)	Area
500	13992.83
400	11877.2
250	6672.44
100	2300.21
75	1789.75
50	1100.86
25	527.44
0	0

Supplement Graph 20. *The standard curve for absorbance vs. concentration of analog Linear-36 utilizing **UV detection** (210 nm).*

d. Series 2 Macrocyclic Analogs: PAMPA Data and Standard Curves

Data has been previously reported.[4]

e. Series 2 Linear Analogs: PAMPA Data and Standard Curves

Acceptor Concentration (uM)					
**L1.4**	**L2.1**	**L2.2**	**L2.3**	**L2.4[a]**	**L2.5**
52.3	83.2	68.3	89.6	56.0	55.5
57.9	94.6	68.9	71.9	58.6	54.4
64.4	90.8	76.7	75.1	59.5	52.1
61.4	98.2	75.8	81.3	58.3	59.0
62.2	93.2	62.9	83.9	60.6	58.4
61.3	76.9	70.1	71.2	61.7	67.8
51.5	74.6	66.3	69.7	63.1	57.4
55.0	97.5	62.1	66.2	60.4	59.8
59.1	85.3	69.0	69.9	60.3	61.8
51.7	83.9	63.9	69.0	61.4	56.8
**Ave: 57.7**	**78.0**	**77.2**	**74.8**	**60.0**	**58.3**
**SD: 4.7**	**3.2**	**5.5**	**7.6**	**2.0**	**4.4**

Supplement Table 17. PAMPA acceptor concentrations (UV detection) for Linear Analogs. *The acceptor concentrations were calculated using a standard curve. The initial concentration is 500 uM for all experiments. The average [acceptor] and standard deviations are listed in the final 2 rows of the table. There were 10 experiments for each analog.*

Papp (x 10^{-6} cm/s)					
**L1.4**	**L2.1**	**L2.2**	**L2.3**	**L2.4[a]**	**L2.5**
1.61	3.57	2.7	3.76	2.33	2.47
2.02	4.24	2.86	3.12	2.56	2.25
2.01	3.98	3.2	3.36	2.52	2.11
2.03	4.55	2.91	3.52	2.51	2.43
2.24	4.01	2.54	3.88	2.64	2.68
2.09	3.22	2.78	3.19	2.55	3.13
1.63	3.32	2.8	3.05	2.62	2.40
1.88	4.45	2.55	2.93	2.64	2.43
2.15	4.33	2.9	3.14	2.69	2.64
1.75	4.04	2.68	3.20	2.67	2.27
**Ave: 1.94**	**3.97**	**2.79**	**3.32**	**2.57**	**2.48**
**SD: 0.22**	**0.46**	**0.19**	**0.31**	**0.11**	**0.29**

Supplement Table 18. PAMPA Papp Values (UV detection) for Linear Analogs. *The Papp were calculated using* ***Supplement Equation 1****. The average Papp and standard deviations are listed in the final 2 rows of the table. There were 10 experiments for each analog.*

[a] L2.4 was subjected to the PAMPA assay as the boc-deprotected product.

Cmpd	MW (g/mol)	Ave RT (min)	C ($x10^{-9}$)	Well 1		Well 2		Well 3		Well 4		Well 5		Ave. %R
				%T	%R	%T	%R	%T	%R	%T	%R	%T	%R	
L1.4	1061.4	11.8	3.23	0.25	94	0.30	87	0.30	99	0.31	93	0.33	87	90.04 ± 4.46
				0.31	90	0.25	92	0.28	87	0.32	85	0.26	86	
L2.1	1060.4	10.9	2.99	0.47	97	0.47	94	0.49	96	0.48	92	0.49	98	93.46 ± 4.54
				0.50	99	0.52	93	0.49	93	0.50	84	0.50	88	
L2.2	1059.4	11.8	2.89	0.34	95	0.40	91	0.36	92	0.36	98	0.38	92	92.16 ± 3.01
				0.40	95	0.40	89	0.39	91	0.38	90	0.40	89	
L2.3	1059.4	9.9	3.04	0.55	96	0.56	91	0.52	89	0.50	92	0.54	87	89.45 ± 3.85
				0.49	88	0.52	90	0.55	89	0.53	88	0.53	85	
L2.4	958.3	9.9	3.19	0.52	91	0.54	87	0.53	89	0.50	88	0.53	87	88.53 ± 2.10
				0.53	92	0.54	92	0.50	90	0.52	86	0.52	88	
L2.5	1057.4	9.6	3.22	0.61	84	0.61	89	0.58	90	0.60	90	0.61	82	87.74 ± 3.81
				0.63	83	0.60	89	0.63	91	0.62	88	0.64	92	

Supplement Table 19. The Variables from Calculations for Linear Analogs. *Analog L1.1 data is comprised of 2 experiments, each with 5 replicates run in tandem (10 total). Analog L1.2 data is comprised of 2 experiments, each with 5 replicates run in tandem (10 total). Analog L1.3 data is comprised of 2 experiments, each with 5 replicates run in tandem (10 total). Analog L1.4 data is comprised of 2 experiments, each with 5 replicates run in tandem (10 total). Analog L1.5 data is comprised of 2 experiments, each with 5 replicates run in tandem (10 total). Analog L1.6 data is comprised of 2 experiments, each with 5 replicates run in tandem (10 total). The molecular weights and mean retention times seen via HPLC (210 nm) are listed. The C values and %T values are calculated using Supplement Equation **1**. The mean percent recoveries are listed. The data shown is only for UV detection. Definitions: MW = molecular weight, RT = retention time, %R = % recovery, Ave. %R = mean ± standard deviation.*

Cmpd	Ave. $logP_{app}$	Ave. logP	Well 1		Well 2		Well 3		Well 4		Well 5		Ave. [acc]	Ave. % diff
			[acc]	[donor]	[acc]	[donor]	[acc]	[donor]	[acc]	[donor]	[acc]	[donor]		
L1.4	-5.71 ± 0.05	-9.04 ± 0.04	52.3	417.0	57.9	378.9	64.4	431.9	61.4	403.7	62.2	372.2	57.69 ± 4.75	11.54 ± 0.95
			61.3	390.2	51.5	406.3	55.0	381.8	59.1	364.0	51.7	379.3		
L2.1	-5.40 ± 0.05	-9.21 ± 0.05	83.2	401.8	94.6	377.4	90.8	388.0	98.2	362.8	93.2	394.9	87.8 ± 8.3	17.56 ± 1.66
			76.9	417.9	74.6	392.7	97.5	368.2	85.3	335.4	83.9	355.8		
L2.2	-5.55 ± 0.03	-9.32 ± 0.03	68.3	406.1	68.9	384.5	76.7	381.3	75.8	416.4	62.9	397.9	68.4 ± 4.9	13.68 ± 0.99
			70.2	404.1	66.3	379.6	62.1	391.5	69.0	380.2	63.9	382.5		
L2.3	-5.48 ± 0.04	-9.28 ± 0.05	89.6	388.1	71.9	381.8	75.1	368.6	81.3	379.7	83.9	352.9	74.8 ± 7.6	14.96 ± 1.53
			71.2	368.8	69.7	380.0	66.2	377.0	69.9	370.0	69.0	358.0		
L2.4	-5.59 ± 0.02	-9.38 ± 0.02	56.0	396.3	58.6	375.4	59.5	386.7	58.3	382.2	60.6	376.5	60.0 ± 2.0	12.00 ± 0.40
			61.7	396.6	63.1	394.6	60.4	374.3	60.3	367.5	61.4	376.6		
L2.5	-5.61 ± 0.05	-9.40 ± 0.03	55.5	361.8	54.4	391.1	52.1	399.5	59.0	391.3	58.4	350.7	58.3 ± 4.4	11.66 ± 0.87
			67.8	344.8	57.4	386.3	59.8	397.4	61.8	375.8	56.8	405.2		

Supplement Table 20. Final Experimental Data for Macrocyclic Analogs. *The mean $logP_{app}$ (calculated from Supplement Equation 1) and logP values (calculated from (Supplement Equation 2) are listed. The acceptor and donor concentrations are calculated using a standard curve and the units are uM. The initial concentration is 500 uM for all experiments. The total mean acceptor concentrations are listed in the final column.). The data shown is only for UV detection. Definitions: [acc] = acceptor concentration after incubation, [donor] = donor concentration after incubation, mean [acc] = mean acceptor concentration ± standard deviation.*

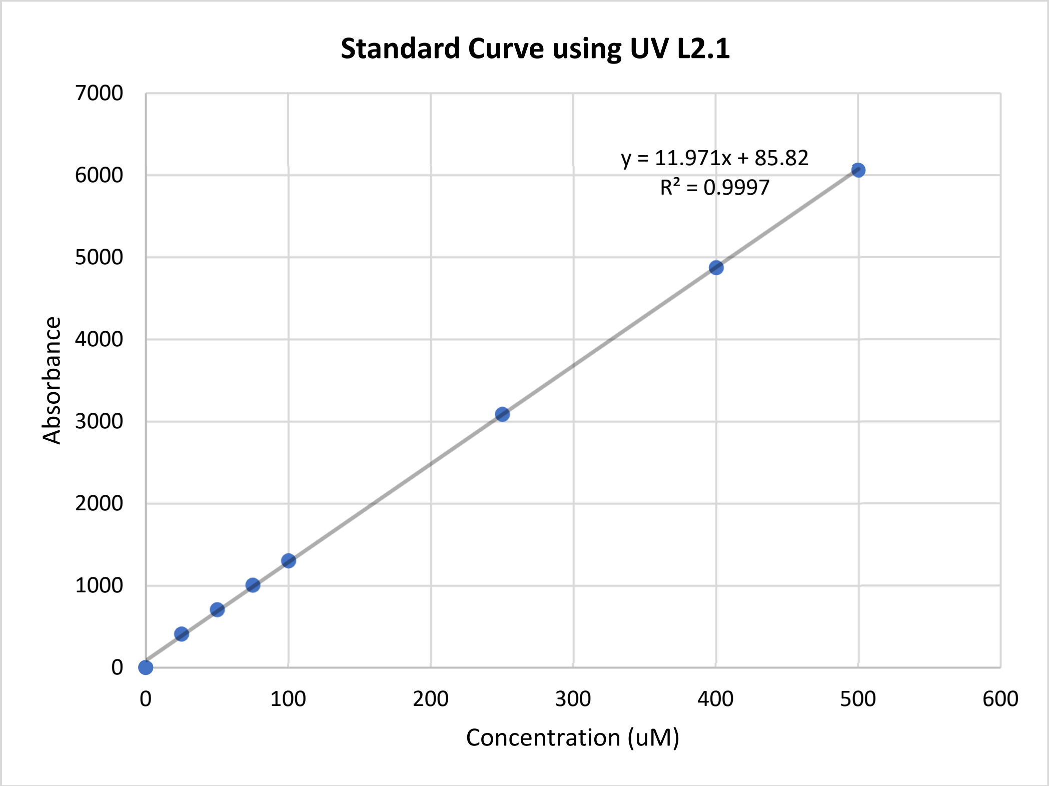

Conc. (uM)	Area
500	6059.33
400	4870.33
250	3086.83
100	1303.33
75	1006.08
50	708.83
25	411.58
0	0

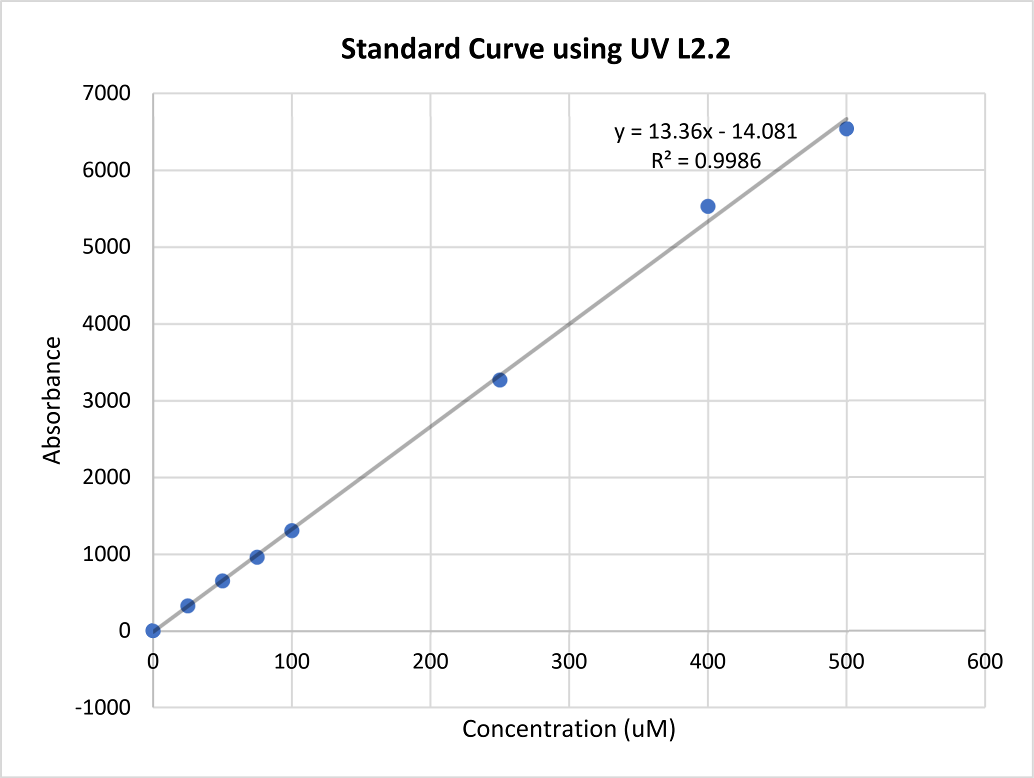

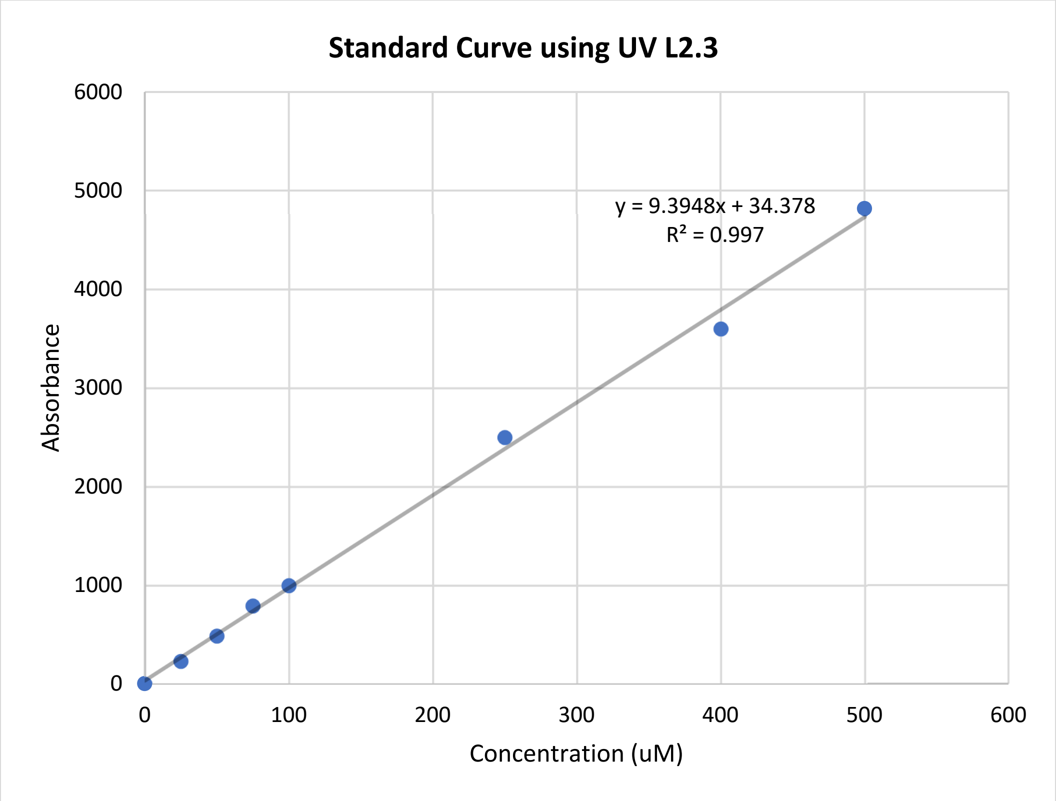

Conc. (uM)	Area
500	4818.3
400	3599.5
250	2499.6
100	[illegible]
75	[illegible]
50	[illegible]
25	[illegible]
0	0

Conc. (uM)	Area
500	6538.9118
400	[illegible]
250	[illegible]
100	[illegible]
75	[illegible]
50	[illegible]
25	327.3368
0	0

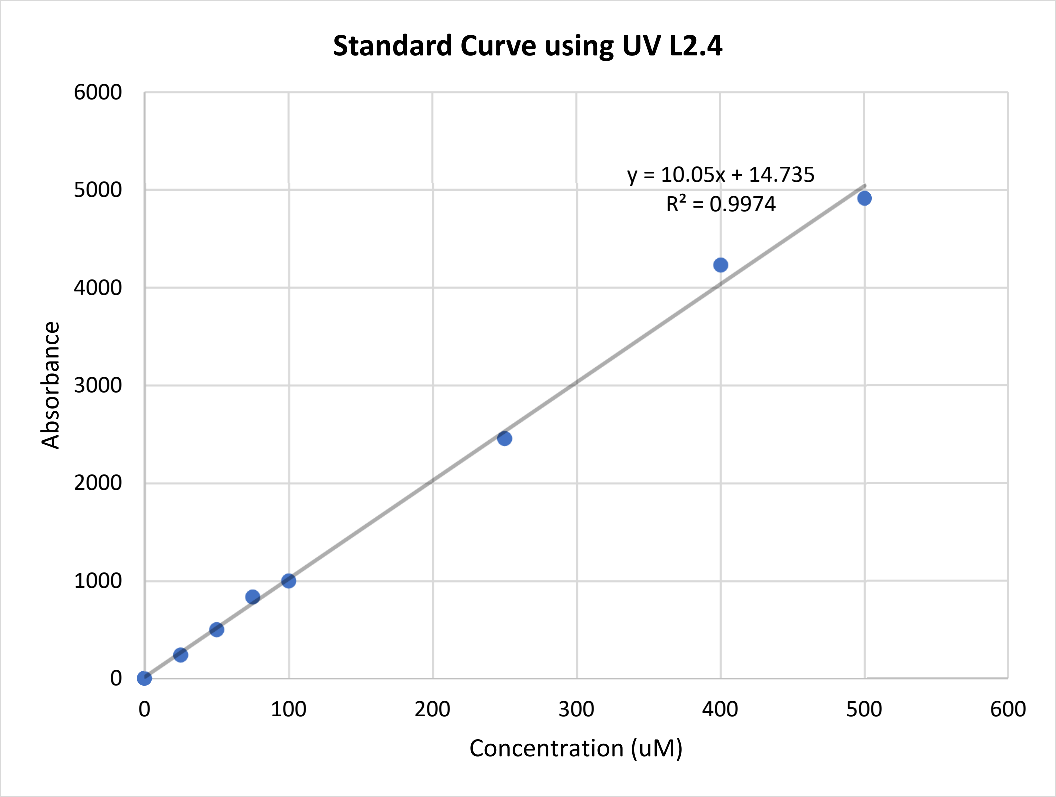

Conc. (uM)	Area
500	4914.4
400	4230.6
250	2456.689
100	1001.29
75	837.6
50	503.11
25	244.59
0	0

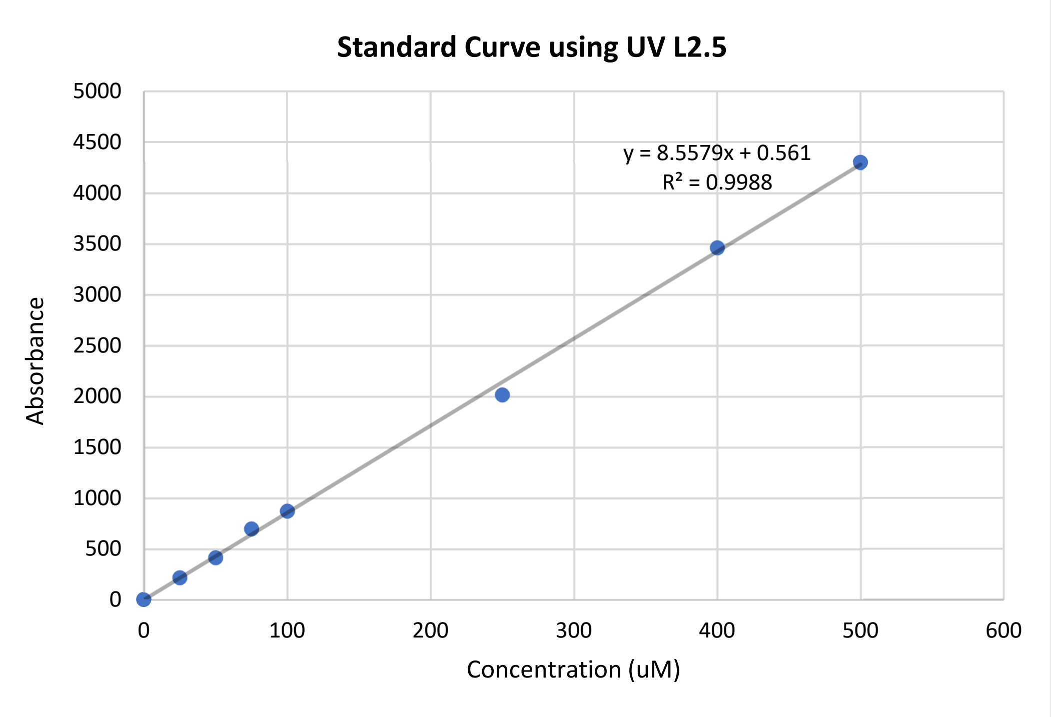

Conc. (uM)	Area
500	4300.1
400	3461.7
250	2016.33
100	873.4
75	699.3
50	415.2
25	219.5
0	0

IV. Statistical Analysis

4.1 Series 1: Ring-Size Analogs

a. Macrocyclic Variants (C1.1-1.6)

$LogP_{app}$			Acceptor Concentration		
Pair	P-value	Inference	Pair	P-value	Inference
A vs B	0.001	**	A vs B	0.001	**
A vs C	0.001	**	A vs C	0.001	**
A vs D	0.001	**	A vs D	0.001	**
A vs E	0.001	**	A vs E	0.001	**
A vs F	0.001	**	A vs F	0.001	**
B vs C	0.025	*	B vs C	0.060	NS
B vs D	0.169	NS	B vs D	0.001	**
B vs E	0.011	*	B vs E	0.001	**
B vs F	0.001	**	B vs F	0.001	**
C vs D	0.001	**	C vs D	0.001	**
C vs E	0.899	NS	C vs E	0.001	**
C vs F	0.001	**	C vs F	0.001	**
D vs E	0.001	**	D vs E	0.001	**
D vs F	0.001	**	D vs F	0.001	**
E vs F	0.104	NS	E vs F	0.001	**

Supplement Table 21. Data Analysis of Series 1 Macrocyclic Analogs *Kruskal-Wallis test with Dunn's multiple comparisons post hoc analysis using https://astatsa.com/OneWay_Anova_with_TukeyHSD/_result/. Definitions: A = C1.1, B = C1.2, C = C1.3 (ent-18), D = ent-verticilide (C1.4), E = C1.5, F = C1.6 and ** p < 0.01, * p < 0.05, NS = not significant.*

b. Linear Variants (L1.1-1.6)

$LogP_{app}$			Acceptor Concentration		
Pair	P-value	Inference	Pair	P-value	Inference
A vs B	0.001	**	A vs B	0.001	**
A vs C	0.001	**	A vs C	0.001	**
A vs D	0.001	**	A vs D	0.001	**
A vs E	0.001	**	A vs E	0.001	**
B vs C	0.899	NS	B vs C	0.577	NS
B vs D	0.001	**	B vs D	0.001	**
B vs E	0.001	**	B vs E	0.001	**
C vs D	0.001	**	C vs D	0.001	**
C vs E	0.001	**	C vs E	0.001	**
D vs E	0.899	NS	D vs E	0.001	**

Supplement Table 22. Data Analysis of Series 1 Linear Analogs *Kruskal-Wallis test with Dunn's multiple comparisons post hoc analysis using https://astatsa.com/OneWay_Anova_with_TukeyHSD/_result/. Definitions: A L1.1, B = L1.2, C = L1.3, D = L1.4, E = L1.5, F = L1.6 and ** p < 0.01, * p < 0.05, NS = not significant.*

4.2 Series 2: Backbone Composition Analogs

a. Macrocyclic Variants (C2.1-2.5)

Data has been previously reported.[4]

b. Linear Variants (L2.1-2.5)

$LogP_{app}$			Acceptor Concentration		
Pair	P-value	Inference	Pair	P-value	Inference
A vs B	0.001	**	A vs B	0.001	**
A vs C	0.001	**	A vs C	0.001	**
A vs D	0.001	**	A vs D	0.001	**
A vs E	0.001	**	A vs E	0.899	NS
A vs F	0.001	**	A vs F	0.899	NS
B vs C	0.001	**	B vs C	0.001	**
B vs D	0.001	**	B vs D	0.001	**
B vs E	0.001	**	B vs E	0.001	**
B vs F	0.001	**	B vs F	0.001	**
C vs D	0.001	**	C vs D	0.899	NS
C vs E	0.523	NS	C vs E	0.021	*
C vs F	0.162	NS	C vs F	0.001	**
D vs E	0.001	**	D vs E	0.001	**
D vs F	0.001	**	D vs F	0.001	**
E vs F	0.899	NS	E vs F	0.899	NS

Supplement Table 23. Data Analysis of Series 2 Linear Analogs *Kruskal-Wallis test with Dunn's multiple comparisons post hoc analysis using https://astatsa.com/OneWay_Anova_with_TukeyHSD/_result/. Definitions: A = L1.4, B = L2.1, C = L2.2, D = L2.3, E = L2.4, F = L2.5 and ** p < 0.01, * p < 0.05, NS = not significant.*

V. Calculated Data – Schrodinger QikProp

The QikProp module (Qikpro 4.2) of Schrödinger was used for the prediction of Absorption, Distribution, Metabolism, Elimination (ADME) properties of all synthesized compounds. The relevant data are tabulated below. Key features include the number of ester bonds (#ester), number of amide bonds (#amide), number of non-trivial (not CX_3) and non-hindered (not alkene, amide, small ring) rotatable bonds (#rotor), molecular weight in g/mol (MW), predicted octanol/water partition coefficient ($QPlogP_{o/w}$), predicted apparent Caco-2 cell permeability in nm/sec (QPPCaco), predicted apparent MDCK cell permeability in nm/sec (QPPMDCK), and Van der Waals surface area of polar nitrogen+oxygen atoms (PSA). *This software can be purchased online.*
https://www.schrodinger.com/platform/products/qikprop/

Entry ID	#ester	#amide	#rotor	MW	$QPlogP_{o/w}$	QPPCaco	QPPMDCK	PSA
**C1.1**	1	1	4	213.276	0.380	671.995	568.849	66.634
**C1.2**	2	2	8	426.552	1.958	407.466	577.299	100.707
**C1.3**	3	3	24	639.828	2.912	522.901	417.555	151.688
**C1.4**	4	4	32	853.104	2.628	107.980	112.155	211.917
**C1.5**	5	5	40	1066.380	3.255	67.246	78.612	250.893
**C1.6**	6	6	48	1279.656	4.050	31.362	57.324	288.040
**L1.1**	1	0	11	421.533	5.169	1752.163	907.037	89.261
**L1.2**	2	1	19	634.809	5.513	775.737	375.962	142.130
**L1.3**	3	2	27	848.085	6.925	289.464	240.490	187.354
**L1.4**	4	3	35	1061.361	6.718	40.932	23.470	242.722
**L1.5**	5	4	43	1274.637	8.910	75.412	76.915	296.928
**L1.6**	-	-	-	-	-	-	-	-

Entry ID	#ester	#amide	#rotor	MW	$QPlogP_{o/w}$	QPPCaco	QPPMDCK	PSA
**C2.1**	3	5 (1 N-H)	32	852.119	2.899	190.315	252.313	203.212
**C2.2**	3	6 (2 N-H)	32	851.134	2.659	171.891	324.761	207.072
**C2.3**	2	6 (2 N-H)	32	851.134	3.071	29.503	89.81	202.936
**C2.4**	1	7 (3 N-H)	32	850.15	2.061	62.133	188.681	210.084
**C2.5**	0	8 (4 N-H)	32	849.165	1.998	160.985	432.205	214.186
**L2.1**	3	5 (1 N-H)	35	1060.376	7.180	58.171	60.354	252.34
**L2.2**	2	6 (2 N-H)	35	1059.391	6.880	55.305	70.399	249.235
**L2.3**	2	6 (2 N-H)	35	1059.391	6.914	42.989	64.888	246.351
**L2.4**	1	7 (3 N-H)	34	958.289	3.774	42.329	71.442	238.121
**L2.5**	0	8 (4 N-H)	35	1057.421	6.574	24.850	86.229	253.309

5.1 Comparison of Experimental Caco-2 Papp to Calculated LogP

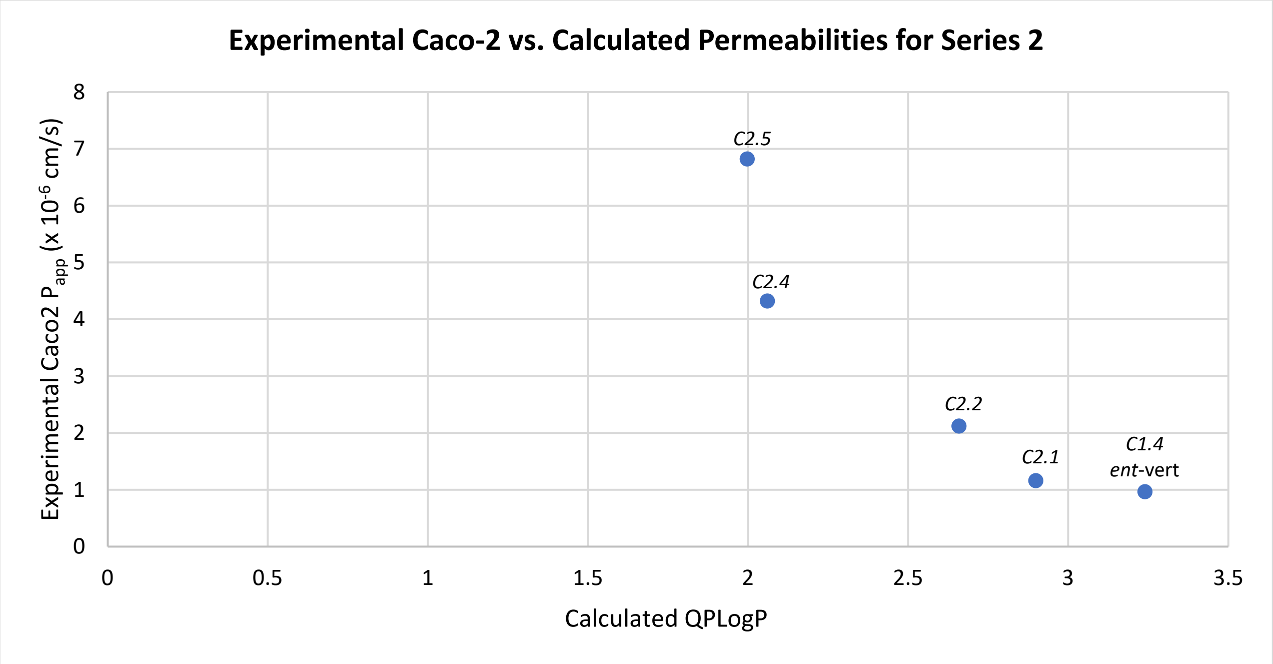

*C2.3 was not detected in Caco-2 experiments

VI. LC/MS and ELSD Traces

In all cases, the large peak near the beginning of the run (R.T. 2-6 minutes), is the DMSO solvent front.

6.1 Macrocyclic Series

a. Series 1

C1.1

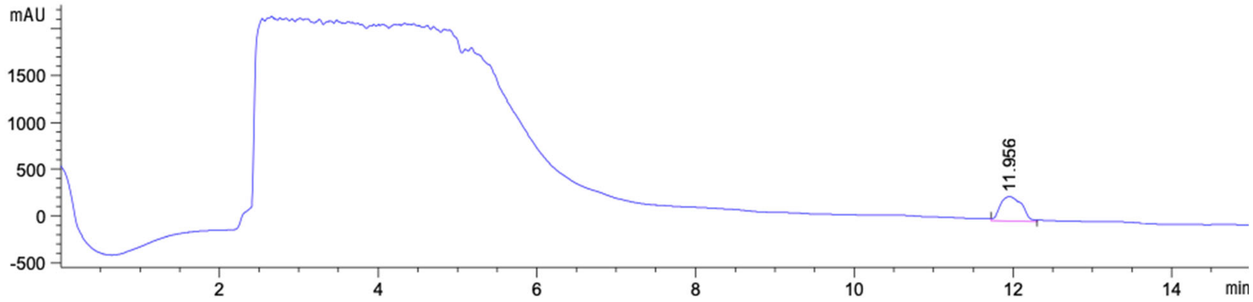

C1.2

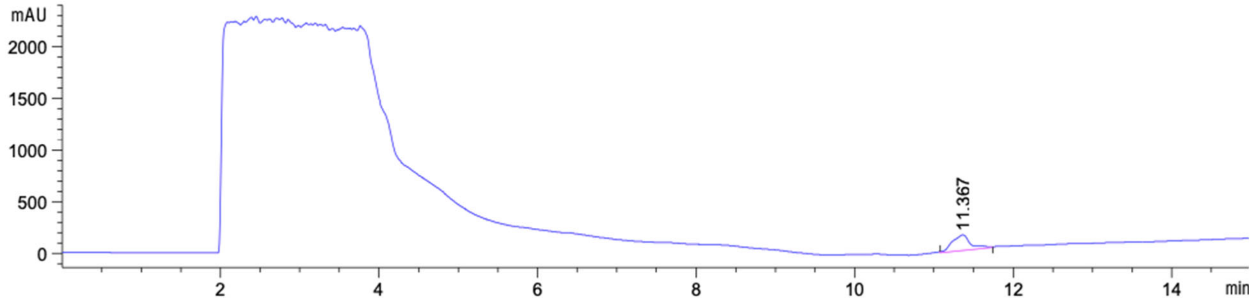

C1.3

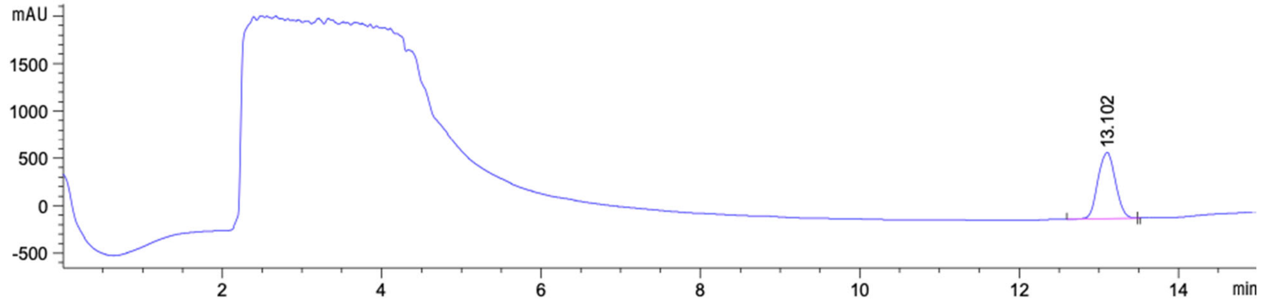

ent-Verticilide (C1.4)

Traces for ent-verticilide (C1.4) were previously reported.4

C1.5

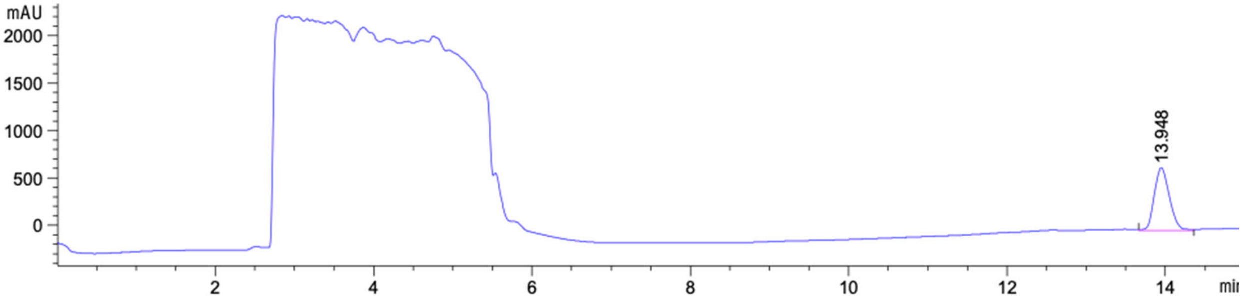

C1.6

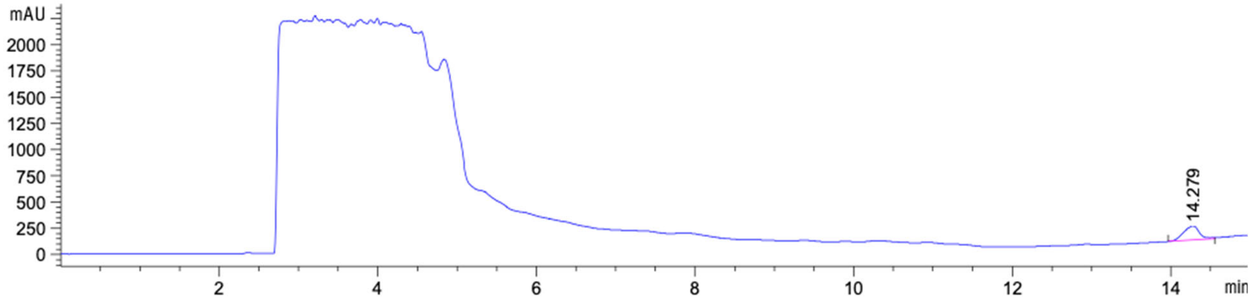

b. Series 2

Traces for C2.1, C2.2, C2.3, C2.4, and C2.5 were previously reported.4

6.2 Linear Series

a. Series 1

L1.1

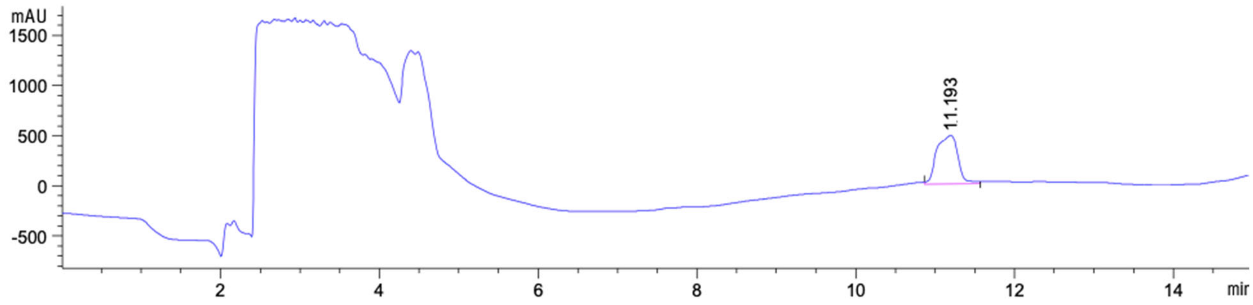

L1.2

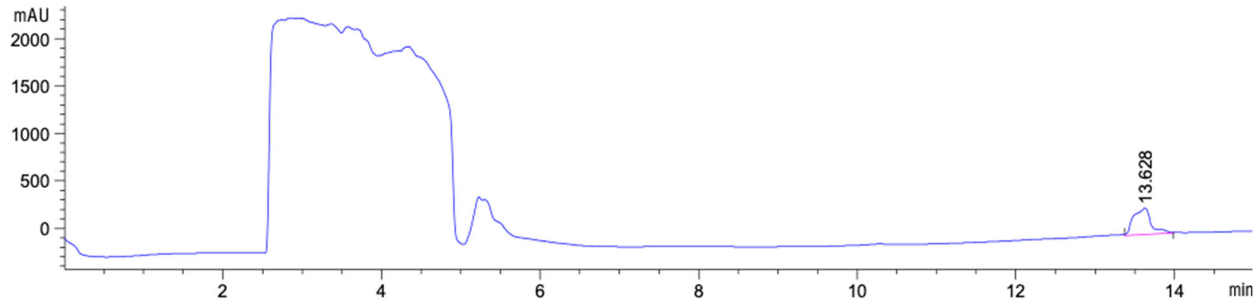

L1.3

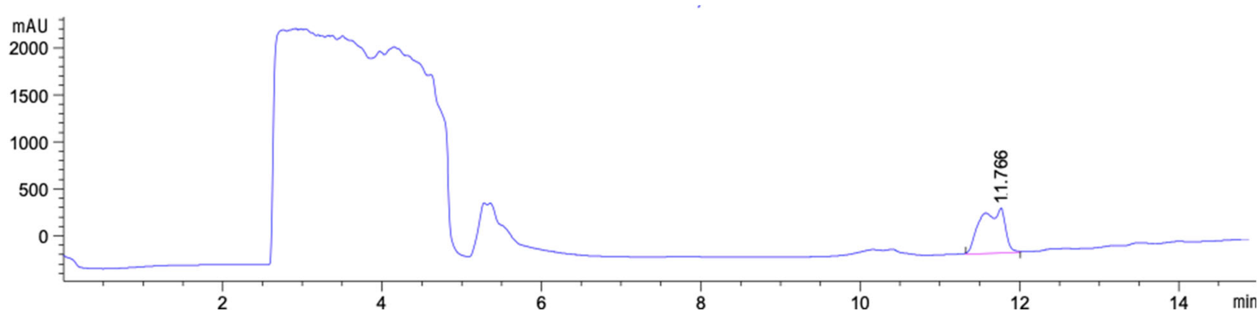

L1.4

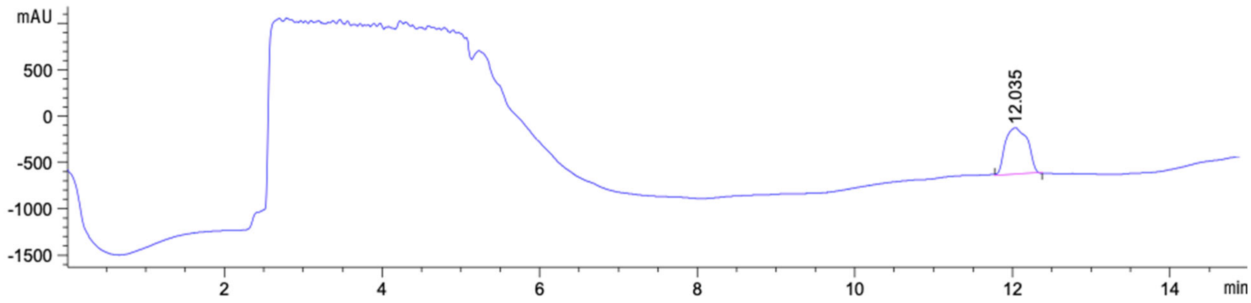

L1.5

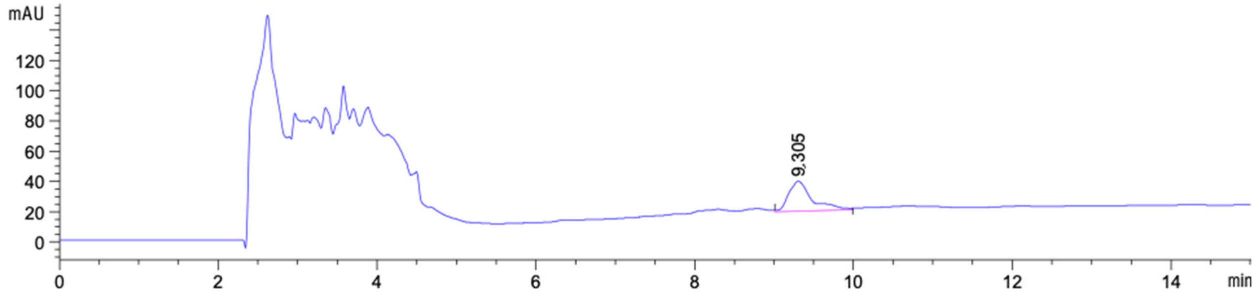

L1.6

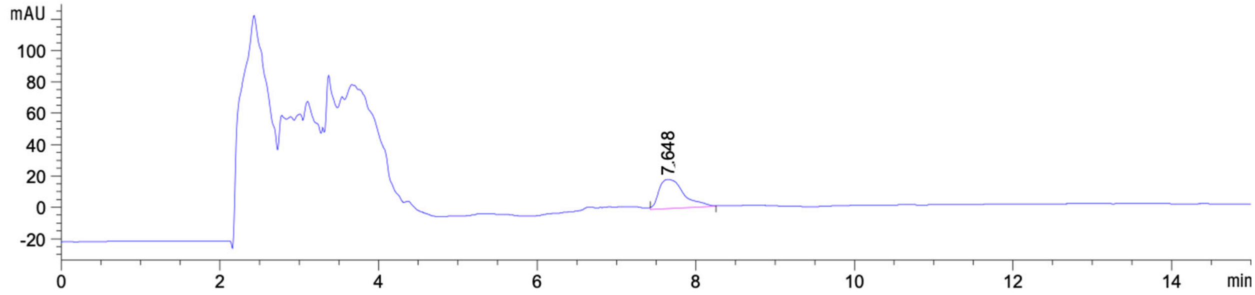

b. Series 2

L2.1

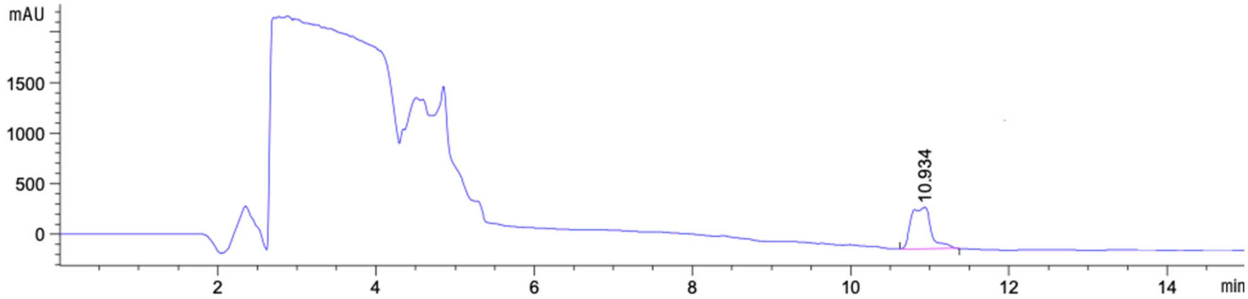

L2.2

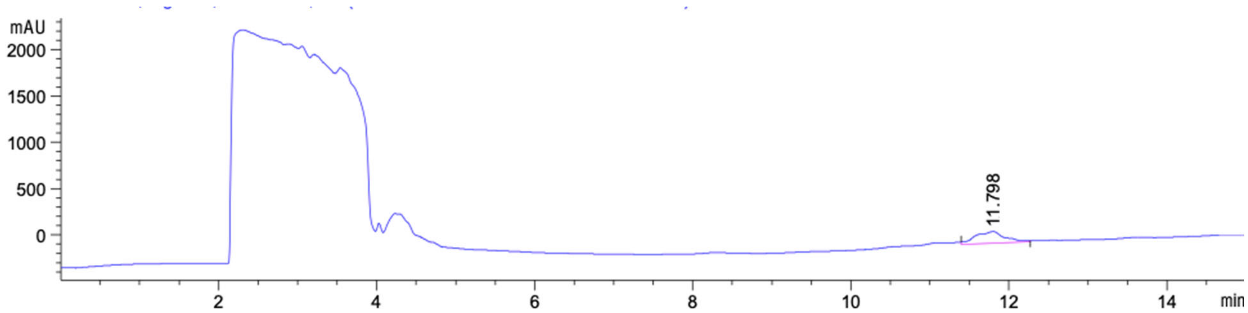

L2.3

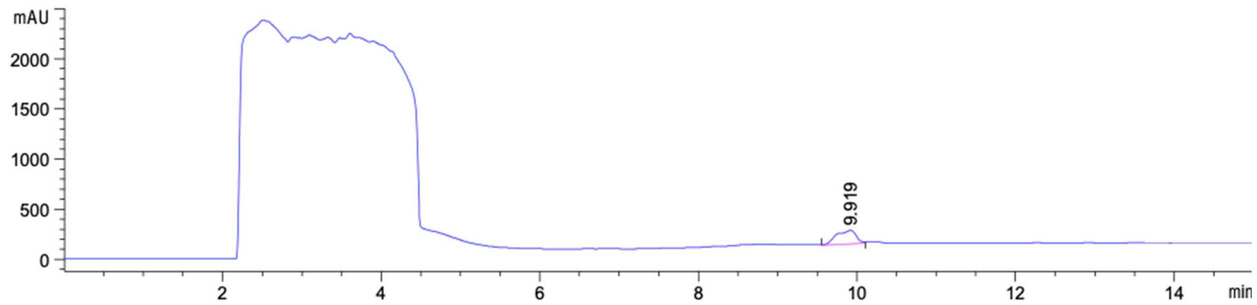

L2.4

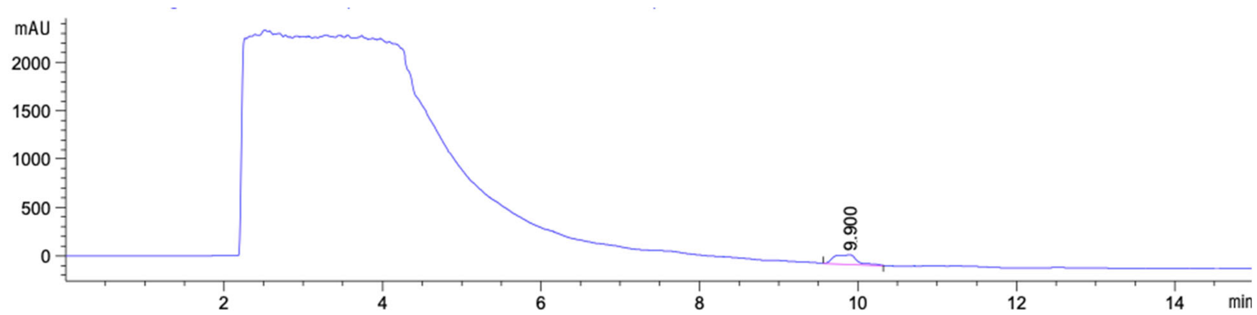

L2.5

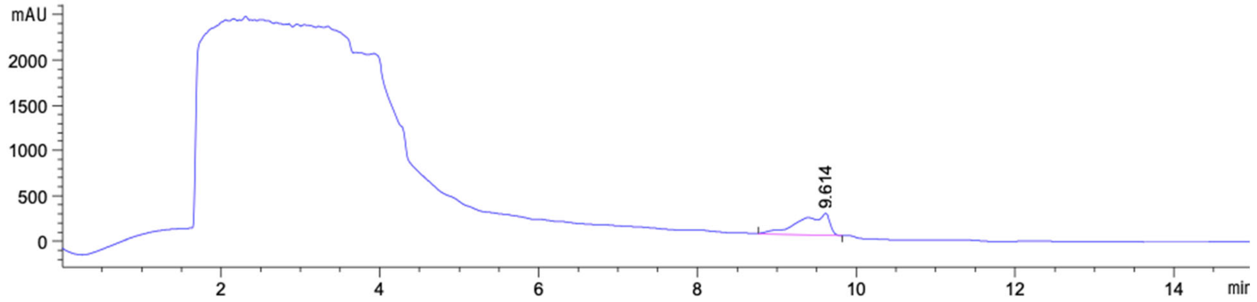

---

[1] Pangborn, A. B.; Giardello, M. A.; Grubbs, R. H.; Rosen, R. K.; Timmers, F. J. Safe and convenient procedure for solvent purification, *Organometallics* **1996**, *15*, 1518.
[2] Šeflová, J.; Schwarz, J. A.; Smith, A. N.; Svensson, B.; Blackwell, D. J.; Phillips, T. A.; Nikolaienko, R.; Bovo, E.; Rebbeck, R. T.; Zima, A. V.; Thomas, D. D.; Van Petegem, F.; Knollmann, B. C.; Johnston, J. N.; Robia, S. L.; Cornea, R. L. RyR2 Binding of an Antiarrhythmic Cyclic Depsipeptide Mapped Using Confocal Fluorescence Lifetime Detection of FRET, *ACS Chem. Biol.* **2023**, *18*, 2290.
[3] Smith, A. N.; Blackwell, D. J.; Knollmann, B. C.; Johnston, J. N. Ring Size as an Independent Variable in Cyclooligomeric Depsipeptide Antiarrhythmic Activity, *ACS Med. Chem. Lett.* **2021**, *12*, 1942.
[4] Thorpe, M. P.; Smith, A. N.; Blackwell, D. J.; Hopkins, C. R.; Knollmann, B. C.; Akers, W. S.; Johnston, J. N. The backbone constitution drives passive permeability independent of side chains in depsipeptide and peptide macrocycles inspired by ent-verticilide, *Chem. Sci.* **2024**, *15*, 14977.
